# Uncommon and nonpartisan: Antidemocratic attitudes in the American public

**DOI:** 10.1073/pnas.2313013121

**Published:** 2024-03-18

**Authors:** Derek E. Holliday, Shanto Iyengar, Yphtach Lelkes, Sean J. Westwood

**Affiliations:** ^a^Department of Political Science, Stanford University, Stanford, CA 94305; ^b^Annenberg School for Communication, University of Pennsylvania, Philadelphia, PA 19104; ^c^Department of Government, Dartmouth College, Hanover, NH 03755

**Keywords:** democratic backsliding, democratic norms, polarization

## Abstract

Democratic regimes flourish only when there is broad acceptance of an extensive set of norms and values. In the United States, fundamental democratic norms have recently come under threat from prominent Republican officials. We investigate whether this antidemocratic posture has spread from the elite level to rank-and-file partisans. Exploiting data from a massive repeated cross-sectional and panel survey (n = 45,095 and 5,231 respectively), we find that overwhelming majorities of the public oppose violations of democratic norms, and virtually nobody supports partisan violence. This bipartisan consensus remains unchanged over time despite high levels of affective polarization and exposure to divisive elite rhetoric during the 2022 political campaign. Additionally, we find no evidence that elected officials’ practice of election denialism encourages their constituents to express antidemocratic attitudes. Overall, these results suggest that the clear and present threat to American democracy comes from unilateral actions by political elites that stand in contrast to the views of their constituents. In closing, we consider the implications of the stark disconnect between the behavior of Republican elites and the attitudes of Republican voters.

## Significance

While American political elites increasingly exhibit an antidemocratic posture, our analysis of public attitudes reveals a clear democratic disconnect: Democrats and Republicans overwhelmingly and consistently oppose norm violations and partisan violence–even when their own representatives engage in antidemocratic actions. This commitment to democratic norms remains stable over time in both cross-sectional and panel data, suggesting that recent outbreaks of antidemocratic behavior on the part of political elites have yet to weaken the public’s support for democracy. However, a more ominous implication of our findings is that public support is not a prerequisite for elite backsliding.

There is general agreement that American democracy is under threat (1, 2). Prominent Republican politicians undermined and attempted to overthrow the 2020 election and many Republican-controlled state legislatures enact policy agendas associated with democratic backsliding (3). Former President Trump, who was already impeached for abusing power and obstructing Congress, promised not to be a dictator—“Except for day one” (4). However, it is important to note that the lack of commitment to democratic norms is not exclusively a Republican phenomenon. Democratic elites also deny the legitimacy of election outcomes (5, 6), and many policies supported by prominent democratic politicians–including “packing” the Supreme Court, (7) and using executive orders to circumvent Congress (8)–are also derided as antidemocratic. What remains unclear, however, is whether and to what extent the actions of leaders represent their supporters’ preferences and whether public support for antidemocratic behavior concentrates in one political party.

We address these questions through massive repeated cross-sectional surveys focusing on support for antidemocratic actions and the use of political violence. The surveys ran over a period of 12 mo encompassing the end of the 2022 campaign.

Despite clear antidemocratic posturing on the part of some elected officials, overwhelming majorities of voters from both parties oppose norm violations, and virtually no respondents support political violence. This resounding endorsement of democratic norms occurs despite high levels of affective polarization and dramatic overestimation of opponents’ support for norm violations–both of which are manifested uniformly across parties. While there is some support for norm violations, respondents typically endorse only one of the multiple norm violations included in the survey, with the supported violation varying widely between respondents. Thus, support for specific antidemocratic positions does not seem to derive from a general antidemocratic framework.

Contrary to expectations (9, 10), we find a clear disconnect between elite behavior and public opinion. Our results reveal a rare common ground: Commitment to democratic norms is not a matter of partisanship. As such, public opinion—often condemned by scholars as susceptible to elite manipulation (11, 12)—stands in contrast to the antidemocratic impulses of elites. Even among traditionally influential or stereotypically extreme constituencies (e.g., MAGA Republicans), support for antidemocratic norms is at most ambivalent. However, our results also show that antidemocratic elite behavior can and does occur even when not supported by the citizenry—public opinion does not constrain elected officials from election denialism and similar antidemocratic actions. In an important contradiction, Americans support the tenets of democracy broadly but are nonetheless willing to support elected officials who do not.

We proceed as follows: We first document the level of support for democratic norms, and their purported precursors, in general and by party. We then look at the antidemocratic sentiments of key voting constituencies that might embolden elected officials.

## 1. Norm Violations and American Politics

Recent history illustrates that some American politicians are more than willing to violate democratic norms for political gain. President Donald Trump openly questioned the legitimacy of the 2020 presidential election, called for the “termination” of the Constitution, encouraged insurrectionist behavior, and in doing so was joined by many Republican colleagues (13). Similar actions have been promoted by many Republican members of Congress, who voted to overturn the 2020 election, slowed executive appointments, and routinely leveraged debt ceiling extensions as bargaining tools (3). As elite discourse typically exerts a strong influence on public opinion (9), these actions by Republican elites have naturally raised concerns that the opinions of Republican voters will follow suit (2), though there is disagreement over the extent of such opinion leadership (14, 15). Based on this body of research, we can anticipate that the partisan asymmetry observed among elites should be mirrored in the general public, with Republicans being more accepting of antidemocratic behavior than Democrats.

While scholarship has assessed the American public’s support for democracy (16), the degree to which public support for democratic norms is asymmetric across parties remains largely unknown and widely debated in the literature. Here, we operationalize asymmetric support for democracy as partisan differences in support for various norms related to democracy as well as support for the use of political violence.

### 1.1. Suggestions that Support Is Asymmetric.

A number of experimental studies have found significant asymmetries in partisan support for democracy. While both Democrats and Republicans showed willingness to endorse antidemocratic actions, Republicans were overall more willing than Democrats (17). Republicans are indifferent to copartisan candidates’ endorsement of democratic norm violations such as shutting down Congress or ignoring court decisions, whereas Democratic respondents punished their candidates taking such positions (18). Support for antidemocratic behavior among Republicans is more strongly associated with beliefs that opposing partisans’ are willing to violate democratic norms, a misperception expressed more frequently by Republicans than Democrats in prior work (19), although other studies find that such misperceptions are symmetric across party lines (20). Finally, high levels of support for antidemocratic sentiment exist among Republicans (although this paper did not examine support among Democrats) (21).

As previously noted, an expectation of asymmetric support for democratic norms can also be deduced from the public opinion literature on cue-taking and opinion leadership (9, 22). As Republican elites have proven more likely to support and enact antidemocratic policies in recent years, we might expect their supporters to fall in line.

### 1.2. Suggestions that Support Is Symmetric.

Some studies suggest that support for democracy is symmetric across parties. This work shows that the baseline level of support for norm violations is minimal (23, 24), and any asymmetry in value disagreement is limited to the most strongly sorted of partisans (25). Indeed, such value disagreement may itself be overstated, as authoritarian worldviews are present in extremists on both the ideological left and right (26, 27). Members of both parties rationalize antidemocratic actions by copartisans as pro-democratic through a variety of cognitive biases (28). These studies suggest that any asymmetry in support, while potentially significant in relative terms, will be small in absolute terms.

While elites may influence voter positions on some policy issues (22), there are clear limits to opinion leadership (29). For instance, the ideological preferences of elected officials are far more extreme than the preferences of the mass public (30). Similarly, Americans’ commitment to democratic principles appears resistant to rhetorical appeals to the contrary (31). Americans remain generally committed to procedural fairness (32, 33) and are also generally unwilling to support rules changes that disproportionately benefit their own party (34). Based on this evidence, we might expect asymmetry in support for specific antidemocratic practices but not an asymmetry in diffuse support for democracy itself.

Finally, the game-theoretic literature predicts that support for norm violations will increase symmetrically regardless of the asymmetry in partisan extremism. Polarization drives democratic backsliding because voters become less likely to punish same-party incumbents for norm violations when the challenger is too ideologically distant (35). Despite greater ideological extremism on the right than left, it is the absolute distance between the parties that drives support for democratic norm violations. Both parties, therefore, will be equally willing to trade off policy wins for democratic norms.

## 2. Precursors of Democratic Backsliding

If support for democratic norms is asymmetric or on a path to become asymmetric, we would expect a parallel asymmetry in two purported antecedents of antidemocratic attitudes: affective polarization (2) and exaggerated estimates of the other sides’ antidemocratic tendencies (20, 36).

### 2.1. Affective Polarization.

In the case of affective polarization, recent work suggests that the affectively polarized may be particularly responsive to elite cues (2) and that partisan affect even transmogrifies into antidemocratic attitudes (37), though some scholars have questioned the causal nature of this relationship (23). These studies imply that a necessary precondition for partisan asymmetry in antidemocratic attitudes is a corresponding asymmetry in affective polarization.

### 2.2. Misperceptions.

While misperceptions of opposing partisans’ support for antidemocratic behavior can be self-fulfilling, (20, 36), it is not clear whether such misperceptions should be asymmetric. On the one hand, given the asymmetric behavior by elites, we might expect an asymmetry among ordinary citizens–but in the opposite direction. It is possible that Democrats and not Republicans are more willing to violate norms so as to defend against perceived threats from Republicans. On the other hand, to the extent Republicans have a more exaggerated sense of threat posed by the Democrats, we would anticipate asymmetry in the opposite direction. Hence, as a test of a potential mechanism underpinning any partisan differences in support for antidemocratic action, we also examine whether Republicans and Democrats hold asymmetric views of the antidemocratic threat posed by their opponents.

## 3. Data

We use a nationally representative survey fielded on a total of 64,000 respondents (recruited from the YouGov panel) between September 15, 2022, and October 3, 2023 (38). Every week, the survey fields responses from 1,000 respondents using a common battery of survey items related to affective polarization, democratic norm violations, and political violence. Because survey inattentiveness can bias measurement of support for violations of democratic norms and political violence (24), we exclude respondents who failed a simple attention check. This yields a final sample of 45,095 respondents ( 39). Additionally, respondents were eligible to be reinterviewed every 3 wk, allowing us to conduct both cross-sectional and panel analysis (5,231 respondents were interviewed twice, and 2,376 were interviewed three or more times). Following standard practice, we code leaners as partisans.

## 4. Results

We present our results sequentially. First, we document support for specific norm violations and political violence overall and by party. We also look at potential party differences in predicted precursors of democratic backsliding (misperceptions of the beliefs of the other side and affective polarization). Second, we examine the relationship between elite support for backsliding and citizen attitudes. Third, we show that elected Republicans are more antidemocratic than key Republican voting constituencies.

### 4.1. Public Support for Antidemocratic Policies and Political Violence.

**Fig. 1.**
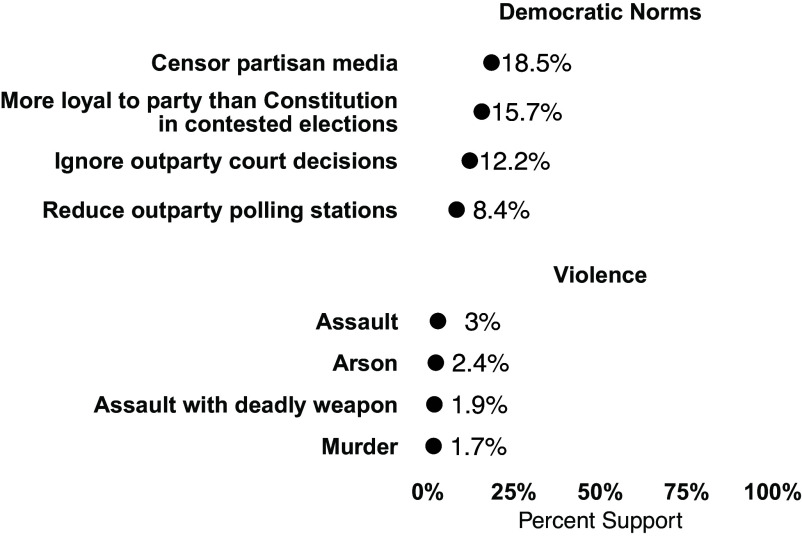
Americans have low levels of support for democratic norm violations and political violence. The 95% CIs are plotted but are generally not visible due to large sample and precise estimates.

We begin by examining aggregate levels of support for democratic norm violations and political violence collapsed across Democrats, Republicans, and Independents in Fig. 1.^*^ In the aggregate, we find a supermajority of Americans in opposition to all norm violations and forms of political violence.^†^

The highest level of aggregate support for norm violations occurs in the case of censoring partisan media (18.5%). This is followed by support for being more loyal to the party than election rules and the Constitution (15.7%), ignoring court decisions that favor the outparty (12.2%) and reducing the number of polling stations in areas dominated by the outparty (8.4%). Turning to the political violence items, we find a decreasing level of support as the action becomes more violent, starting with support for assault (3%), followed by arson (2.4%), assault with a deadly weapon (1.9%), and murder (1.7%).

**Fig. 2.**
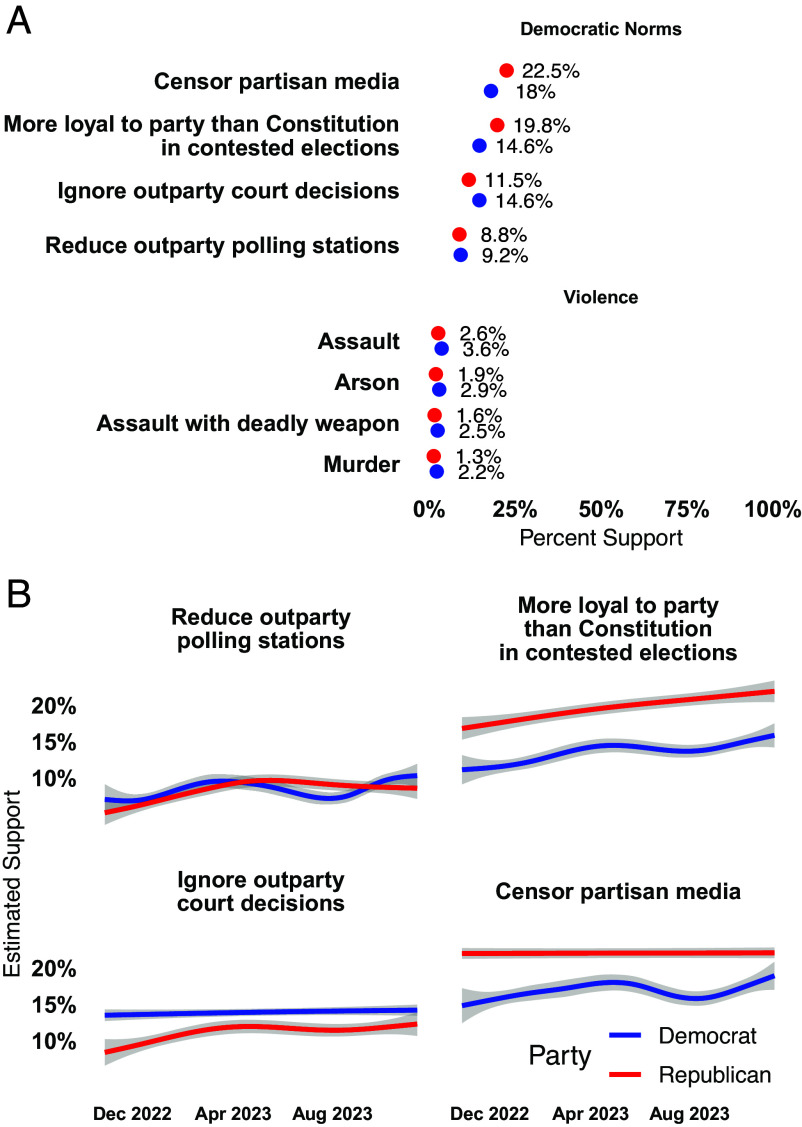
(*A*) Support for norm violations is symmetric. (*B*) Support for norm violations is stable over time. The 95% CIs are plotted but are generally not visible due to large sample and precise estimates.

Public opposition to antidemocratic actions and political violence is not only overwhelming, but also remarkably stable as shown in Fig. 2*B*. Regressing support for democratic norm violations on survey week yields weekly increases in support of only 0.02 to 0.07% across all items, and even lower weekly changes (0.01 to 0.02%) across all support for violence items.^‡^ Using only responses from our panel and including respondent fixed effects yields even weaker trends. Previous responses to both antidemocratic support and political violence items are powerful predictors (substantially and significantly) of future responses.

#### 4.1.1. Bipartisan opposition to democratic backsliding.

We turn to the key question of differences in support for antidemocratic behavior and political violence between partisans in Fig. 2*A*. Across all individual items, a majority of both Democrats and Republicans oppose violations of democratic norms and political violence.

We find no evidence that members of either party have a general antidemocratic disposition. Among partisans who do support antidemocratic actions, a large plurality support only one such violation of democratic norms. Only 17.2% of Democrats and 21.6% of Republicans support one norm violations. The parties have similar levels of support for two violations (6% and 9%) or more.^§^

When looking at individual norms, we observe fairly small partisan differences in support for norm violations. The largest asymmetry is only 5.2 percentage points [95% CI (4.2,6.2)], with Republicans being more likely to support being loyal to the party than the Constitution in contested elections, with other differences ranging between 0.4 [95% CI (−0.4,1.1)] and 4.5 [95% CI (3.5,5.6)] percentage points. Importantly, the party showing the greatest support for norm violations fluctuates, with Democrats and Republicans both leading on two items. Support for specific democratic norms differs but appears far from overwhelmingly asymmetric. These differences pale in comparison to other manifestations of partisan asymmetry in, e.g., sorting and ideological reasoning (40, 41). It is worth acknowledging, however, that Republican respondents show a strong and linear upward trend in support for loyalty to party over the Constitution, the norm for which there is already the most asymmetry in partisan support.

For political violence, the differences between Republicans and Democrats vanish almost entirely. While Democrats are marginally more supportive on all items, this difference peaks at only 1 percentage point [95% CI (0.5,1.5)]. Support within both parties is always below 4% and did not peak around the 2022 midterm.

**Fig. 3.**
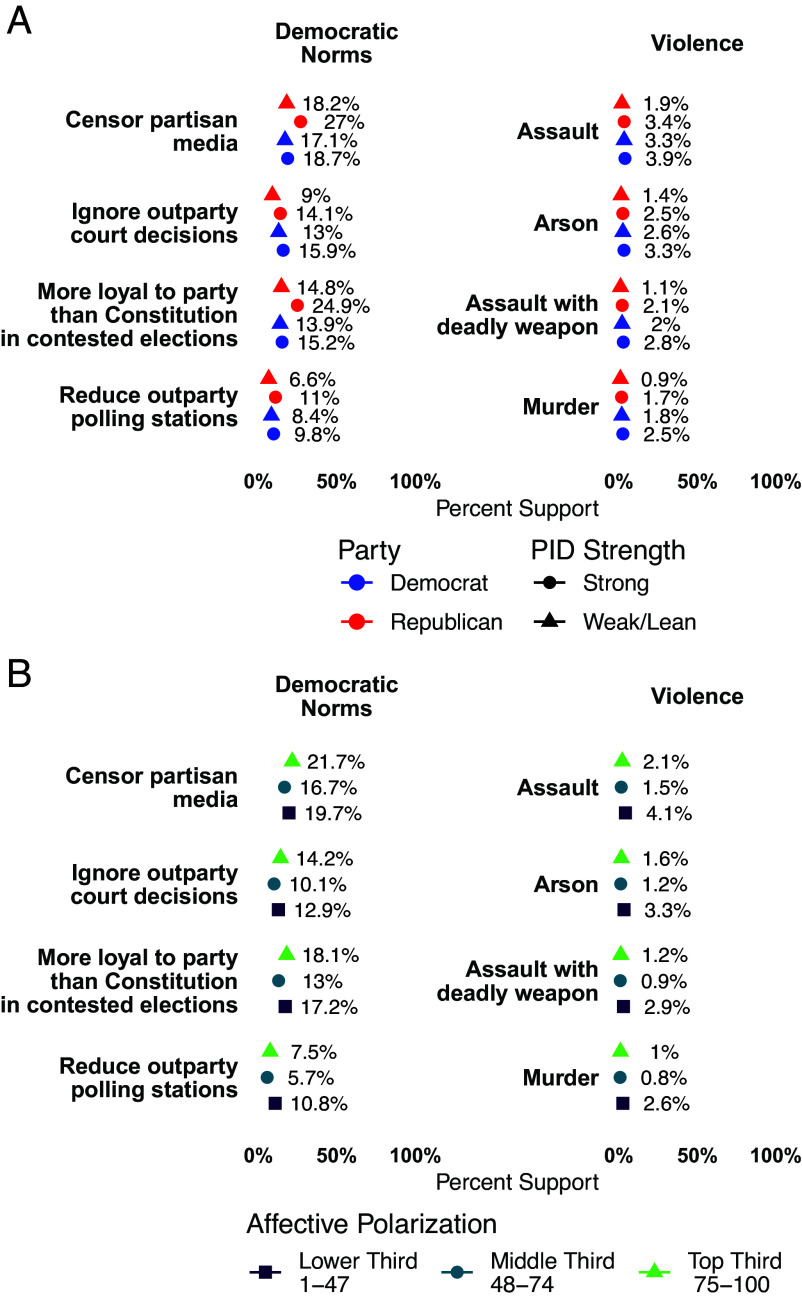
Strength of partisan identity (*A*) and affective polarization (*B*) sometimes barely increase support for norm violations and violence. The 95% CIs are plotted but are generally not visible due to large sample and precise estimates.

Importantly, the relationship between strength of partisanship and our dependent measures is mostly consistent across both parties. Fig. 3*A* shows strong partisans are slightly more likely to endorse norm violations and political violence across all items, but strong Democrats are generally attitudinally similar to strong Republicans. There are, however, more sizable gaps in support for censoring partisan media [8.2 percentage points, 95% CI (6.7,9.8)] and, again, loyalty to party over the Constitution [9.7 percentage points, 95% CI (8.3,11.2)] among strong Republicans and Democrats. So while large majorities of strong partisans still reject democratic norm violations, there is some evidence of asymmetries being driven by strong Republicans, with a particularly strong pattern for loyalty to the party over the Constitution.

#### 4.1.2. Precursors: Affective polarization.

Affective polarization is also symmetric, with an overall difference of 1.4 points [95% CI (0.6,2.2)] between parties (largely attributable to warmer feelings toward their own party by Democrats). Again, these attitudes prove stable over time, with month-to-month variations fluctuating by only one or two points. To the degree there is movement on outparty affect and affective polarization, both parties move together (see *SI Appendix*, section 2 for full model results and figures).

The relationship between terciles of affective polarization and norm violations and political violence is inconsistent as shown in Fig. 3*B*. In some cases, those with most polarized attitudes have the highest support (partisan censorship, loyalty to the party over the Constitution in contested elections), while in other cases support was greatest for those with the lowest levels of affective polarization (reducing outparty polling stations, political violence items). These noisy results are consistent with the work showing the absence of a causal effect of affective polarization on downstream political outcomes (23). Similar to strength of partisan identity, however, the effect is substantively minimal.

#### 4.1.3. Precursors: Misperceptions of the other side.

**Fig. 4.**
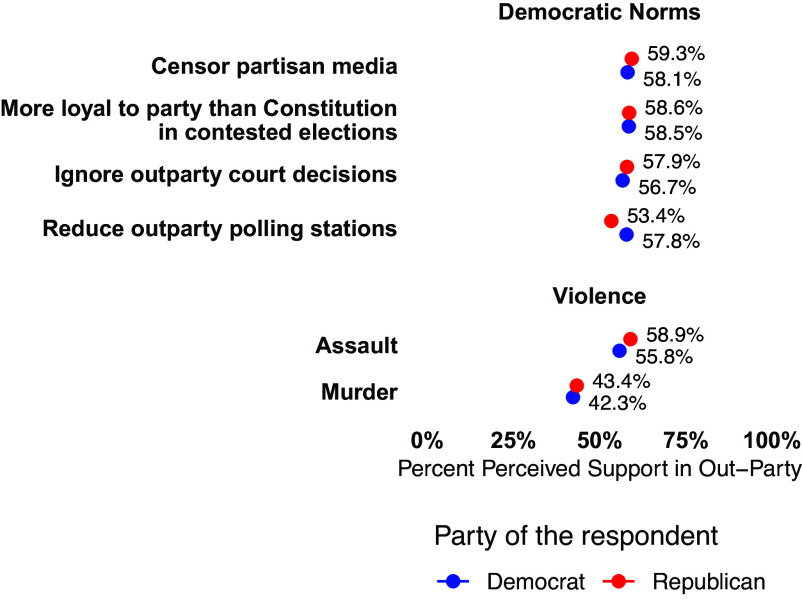
Perceived outparty support for norm violations and violence is high and symmetric. The 95% CIs are plotted but are generally not visible due to large sample and precise estimates.

Negative stereotypes of the outparty could drive expectations of democratic norm violations and violence, which may subsequently drive higher levels of support for such norm violations (36). Consistent with prior work, we find that perceptions of opposing partisans’ support for norm violations exceed actual support, in some cases by four to five times (Fig. 4). However, unlike prior work (42), we find these misperceptions are symmetric. This is likely attributable to our use of measures of norm violations that are severed from the behavior of a former president. This is consistent with game-theoretic models (43), which predict that norm violations should occur at similar rates for both parties.

### 4.2. Policymakers Who Deny Election Outcomes Do Not Reflect the Democratic Preferences of Their Constituents.

Democratic norm violations and political violence are overwhelmingly unpopular in the aggregate, but it is possible that politicians who do support such violations are simply mirroring the views of their constituents. Our results suggest that antidemocratic politicians represent the views of a small minority of their constituency, but the size of that minority might vary by Congressional district.

To test this possibility, we gathered data on U.S. House Representatives who either 1) voted to overturn the 2020 election results (44) or 2) publicly denied the legitimacy of the 2020 election results (45). Then, using a multilevel model with random intercepts for Congressional districts, we assessed whether survey respondents represented by an election-denying Member of Congress were more likely to support being more loyal to the party than to election rules and the Constitution.^¶^

Our dependent variable—loyalty to party—is a binary recoding of support for being more loyal to the party than election rules and the Constitution when a candidate questions the outcome of an election. We include two binary operationalizations of norm violations by House members: “Voted to Overturn” and “Election Denial.” The former reflects votes on January 6th, 2021, to certify the result of the election and the latter indicates whether a respondent’s representative denied the legitimacy of the 2020 election results either through public statements or political action.^#^

As shown in Table 1, the relationship between citizen opinion and elite behavior is neither substantively nor statistically significant. Constituents of representatives who engaged in either form of election denial were no more likely to support loyalty to the party over the Constitution than constituents of representatives who did not engage in such conduct. Republicans are at baseline slightly more likely to support loyalty, but an interaction with party of the representative shows that being a constituent of an election-denying representative does not moderate the base level of Republican support. Objections to the 2020 election occurred more than a year before public data collection began, which makes it possible that support in the public was higher in the period before January 6, 2021. First, we note that if the public mood has changed, representatives have not followed as 157 still deny the presidential election outcome publicly (46). Second, faith in election outcomes among Republicans has not recovered to 2018 levels (77%), and barely moved from 40% in 2020 to 44% in 2022 (47).

**Table 1.** Multilevel regression results

	*Dependent variable:*			
	Public Opinion: Loyalty			
	(1)	(2)	(3)	(4)

MOC Voted to Overturn	−0.006 (0.007)		−0.016 (0.009)	
MOC Election Denial		−0.001 (0.007)		−0.010 (0.008)
Independent Respondent			−0.047^∗∗∗^ (0.006)	−0.046^∗∗∗^ (0.006)
Republican Respondent			0.075^∗∗∗^ (0.005)	0.076^∗∗∗^ (0.005)
Voted to Overturn:Ind.			0.019 (0.012)	
Voted to Overturn:Rep.			−0.003 (0.009)	
Election Denial:Ind.				0.015 (0.012)
Election Denial:Rep.				−0.005 (0.009)
Constant	0.142^∗∗∗^ (0.004)	0.141^∗∗∗^ (0.004)	0.126^∗∗∗^ (0.004)	0.124^∗∗∗^ (0.004)
Observations	40,934	40,934	40,934	40,934
Log Likelihood	−14,991.690	−14,992.020	−14,752.580	−14,753.980

Note: *P*< 0.05; ^∗∗^*P*< 0.01; ^∗∗∗^*P*< 0.001.

Estimated with survey weights and two-sided tests.

SEs in parentheses.

While Republican respondents are more likely to support this particular norm violation, it is important to note the magnitude of the discrepancy between politicians and the broader public. As estimated in Fig. 2, only 19.8% of Republican respondents supported greater loyalty to the party than the Constitution during contested elections. Of the 211 Republicans voting on the certification of election results in January 2021, however, 139 (66%) sustained objections to overturn. The real asymmetry in support for antidemocratic behavior is not between Democratic and Republican voters (where support differs by 5.2 percentage points), but between Republican voters and their elected representatives (where support differs by 46.2 percentage points).

### 4.3. The Most Electorally Potent Groups Have the Lowest Support for Democratic Backsliding.

**Fig. 5.**
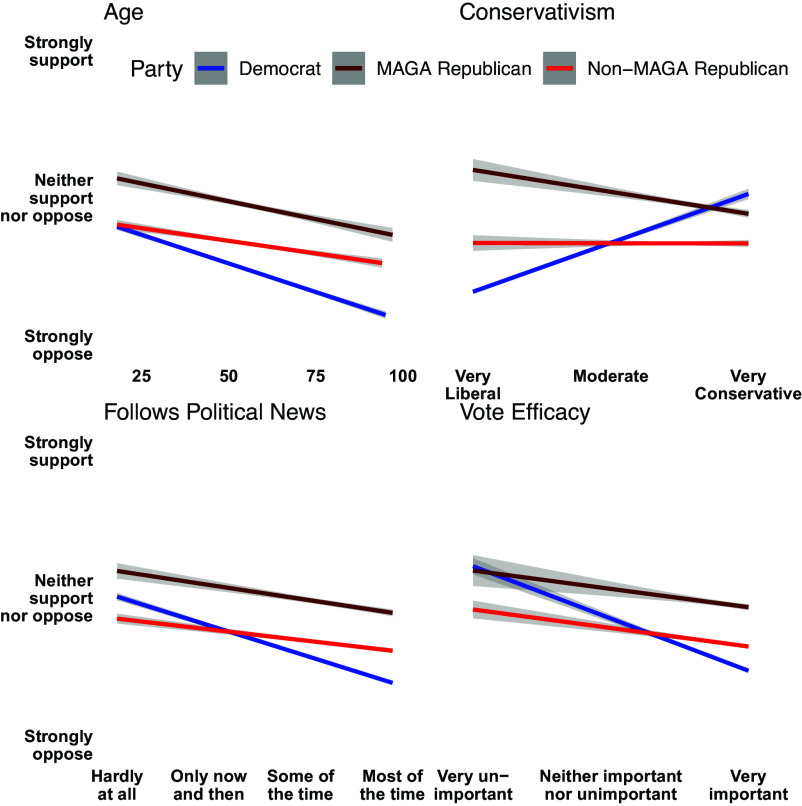
Electorally salient demographics are generally associated with lower support for loyalty to a respondent’s party over the Constitution in contested elections. The 95% CIs are plotted but are generally not visible due to large sample and precise estimates.

It is possible election-denying Republican officials are representing the views of more electorally relevant voting groups, such as primary voters. Such voters in the Republican party tend to be older, more conservative, pay greater attention to politics, and have a higher sense of self-efficacy (48, 49). However, we show in Fig. 5 that voters matching these demographic profiles are generally less likely to support loyalty to the party over the Constitution in contested elections. Even self-identified “MAGA” Republicans are, at most, ambivalent in their antidemocratic attitudes, and their support is moderated by the same factors as non-MAGA Republicans and Democrats.^‖^ The voters such officials claim to represent, then, are neither numerous nor especially influential.

This pattern raises a broader question of which demographic factors best predict antidemocratic attitudes. In *SI Appendix*, section 4, we utilize random forests, a machine-learning approach, to evaluate the importance of different respondent characteristics associated with higher levels of support for democratic norm violations and political violence.

In this analysis, age is the one predictor that is consistently among the most important predictors of support for democratic norm violations. Younger Americans consistently express higher tolerance for norm violations and political violence. For instance, after fitting a simple logistic model of support for ignoring outparty court decisions regressed on age (with survey weights), we predict 21.9% support among 25-y-olds and only 6.8% among 65-y-olds. This is consistent with existing research documenting increasingly polarized preadult socialization (50, 51).

## 5. Discussion

Our results show that Americans of all political stripes overwhelmingly endorse democratic norms and reject the use of political violence. Democrats and Republicans are generally no different in their rejection of norm violations, despite their high levels of negative affect toward each other. We also fail to find party differences in two theorized precursors for support for democratic backsliding: partisan affect and inaccurate perceptions. To the extent asymmetries exist, they exist predominantly in strong Republican support for loyalty to the party over the Constitution in contested elections.

While the recent surge in antidemocratic behavior on the part of American political elites is alarming, our results show these tendencies have yet to filter down to the American public. That Americans have not “followed the leader” on antidemocratic stances is quite remarkable, since the siren song of partisanship has proven so compelling in contemporary politics. While future patterns of support may change (indeed, we already document an increase in support for loyalty to the party over the Constitution over the course of our survey), this disconnect between elite behavior and public opinion suggests the persistence of democratic regime norms among the American public.

More ominous implications of our results are that 1) public support is not a necessary precondition for backsliding behavior by elites, and 2) Americans, despite their distaste for norm violations, continue to elect representatives whose policies and actions threaten democracy. One explanation is that when partisanship is strong, voters place party and policy goals over democratic values (13, 43, 52, 53). Indeed, one of the least supported norm violations—removing polling places in outparty dominated areas—has already been violated by elected officials in Texas (54), and there are concerns about pending similar laws in other states. Such unconstrained elite behavior suggests that threats to democracy could well manifest themselves in both parties in the future. In fact, game theoretic models predict that support for antidemocratic behavior should occur in both parties simultaneously (43) as part of a feedback loop.

Our work is not without limitations. First, our measures are attitudinal, not behavioral (due to sparseness of actual violent behavior and norm violations on the part of ordinary citizens). Second, we did not include a comprehensive set of other predictors that are known to be correlated with norm violations in our analysis. Researchers using a more expansive approach will likely find further success through a more psychological lens accounting for racial attitudes and status threat (55), aggression (24, 56), anti-Establishment attitudes (57), depression (58), and support for authoritarianism (59). Political science often treats these measures as part of the partisanship bundle (60). However, recent work suggests that these traits, while correlated with partisanship, are in fact endogenous to partisan affiliation (61). Pragmatically, many of these measures require large numbers of survey questions and in the face of limited survey time are challenging to record.

Ultimately, the health of American democracy is not contingent on the individual preferences of voters, but the willingness of their elected delegates to abandon the principles of democracy for political gain. The vast majority of Americans do not support democratic norm violations and reject the use of political violence, but these same citizens continue to reelect politicians who are less than fully committed to democracy. Our results suggest that we do not need to convince the public to value democracy, but we do need to convince Americans to take into account their commitment to democracy in the ballot box. If elites can be made more responsive to the attitudes of the public, proponents of democracy will have grounds for cautious optimism.

## 6. Materials and Methods

### 6.1. Question Wording.

Original responses to the following items were given on a five-point scale from strongly agree to strongly disagree. We recode to a binary classification of agree (strongly agree and agree) and not agree (neither agree nor disagree, disagree, and strongly disagree).

Democratic Norms:

1. Reduce outparty polling stations: Do you agree or disagree: (inparty) should reduce the number of polling stations in areas that typically support (outparty).
2. More loyal to party than election rules and constitution: Do you agree or disagree with the following: When a (inparty) candidate questions the outcome of an election other (inparty) should be more loyal to the (inparty) party than to election rules and the constitution.
3. Ignore outparty court decisions: Do you agree or disagree: (inparty) elected officials should sometimes consider ignoring court decisions when the judges who issued those decisions were appointed by (outparty) presidents.
4. Censor partisan media: Do you agree or disagree with the following: The government should be able to censor media sources that spend more time attacking (inparty) than (outparty).
5. President should circumvent congress: Do you agree or disagree: If a (inparty) president can’t get cooperation from (outparty) members of congress to pass new laws, the (inparty) president should circumvent Congress and issue executive orders on their own to accomplish their priorities. Note: We consider this a more minor norm violation, and therefore exclude this measure from the analyses in the main manuscript.

Political Violence:

1. Assault: (name) was convicted of assault. He was arrested by police for throwing rocks at peaceful (outparty) protesters. Although no one was seriously injured, paramedics bandaged a man with a head wound.
  Do you support or oppose (name)’s actions?
2. Arson: (name) was convicted of arson. He was arrested by police as he attempted to run from a fire he started at the local (outparty) headquarters. Although he waited for the building to close for the night, several adjacent buildings were still occupied.
  Do you support or oppose (name)’s actions?
3. Assault with a deadly weapon: (name) was convicted of assault with a deadly weapon. He was arrested by police after driving his car into a crowd of (outparty) protesters. Although no one was killed, several individuals were seriously injured and one spent a month in the hospital.
  Do you support or oppose (name)’s actions?
4. Murder: (name) was convicted of murder. He was arrested by police after surveillance footage was found showing him stabbing a prominent (outparty) to death. (name) targeted the victim because he believed the victim had prevented him from voting in the last election as part of a conspiracy to stop (inparty) voters.
  Do you support or oppose (name)’s actions?

We also field responses for the following electorally salient demographics:

1. MAGA (asked to Republicans only): Would you describe yourself as... (MAGA Republican/Never Trumper/Neither)
2. Vote efficacy: How important or unimportant is it to vote in every election? (Very important/Important/Neither important nor unimportant/Unimportant/Very unimportant)
3. Follows political news: Some people seem to follow what’s going on in government and public affairs most of the time, whether there’s an election going on or not. Others aren’t that interested. Would you say you follow what’s going on in government and public affairs ... (Most of the time/Some of the time/Only now and then/Hardly at all/Don’t know)

### 6.2. Interpretation and Massive Survey Datasets.

Given the large sample size of our survey, our estimates of partisans’ preferences and beliefs are very precise. This level of precision is exceptionally useful for understanding support for antidemocratic behavior at a more granular level and means estimated differences between groups will be equally precise. Specifically, with our sample size, we are powered to detect a standard effect size of 0.03 at significance α=0.05 with 80% frequency. In more tangible terms, take our measure of support for ignoring outparty court decisions as an example. In our sample, a standard effect size of 0.03 would mean a group difference of about 1 percentage point.^**^ We caution readers, however, from inferring substantive significance from statistical significance. It is important to interpret our results within the context of existing literature on democratic backsliding, where the most concern arises when majorities or large pluralities of the population support democratic norm violations or political violence. Of course, as January 6 (or any other act of political terrorism) demonstrated, even a handful of violent Americans can be horrifyingly consequential.

### 6.3. Multilevel Model.

We estimate the following equation for columns 1 and 2 of Table 1:

[1] $$\begin{array}{c} \;{\text{Loyalty}}_{\mathrm{ij}}=\beta_{0ij}+\beta_{1}\;\text{Denier}\;{\text{Rep}}_{\mathrm{ij}}+\epsilon_{\mathrm{ij}}, \end{array}$$

for respondent i in Congressional district j and $\beta_{0ij}$ is a placeholder for separate random intercepts for each district j and respondent i (the latter to account for panelists in our data). A positive coefficient for $\beta_{1}$ suggests respondents being represented by election-denying House members are more supportive of being more loyal to the party than election rules. This would imply some level of responsiveness from a constituency’s representative with regard to democratic norm violations. Of course, one may expect this association to vary by party, as all House members who questioned the legitimacy of the 2020 election were Republicans. We therefore fit the following additional model:

[2] $$\begin{array}{c} \;{\text{Loyalty}}_{\mathrm{ij}}=\beta_{0ij}+\beta_{1}\;\text{Denier}\;{\text{Rep}}_{\mathrm{ij}}+\beta_{2}\;{\text{PID}}_{\mathrm{ij}}+\beta_{3}\;\text{Denier}\;{\text{Rep}}_{\mathrm{ij}}*\;{\text{PID}}_{\mathrm{ij}}+\epsilon_{\mathrm{ij}}, \end{array}$$

where we include an interaction between a respondent having an election-denying representative and the respondent’s party identification. If Republican politicians are responding to greater support for norm violations from their partisan base of support, we should expect the interaction term $\beta_{3}$ for Republicans to be positive (setting Democrats as the reference category).

## Supplementary Material

Appendix 01 (PDF)

## Data Availability

Anonymized CSV data have been deposited in Dataverse. The data and scripts are for the R statistical package. They can be accessed via the following link and https://doi.org/10.7910/DVN/XUTJTN (39).

## Footnotes

^*^Note in this plot and all plots that follow, we show 95% CIs, but the span of the intervals is often so small they are hidden by the size of the points on the plot. All results are weighted to demographic survey targets.

^†^We consider whether this lack of support is a function of social desirability bias in *SI Appendix*, section 6, and find no connection between social desirability (measured on the Marlowe-Crowne Social Desirability Scale) and support for norm violations or violence.

^‡^Results of these analyses are presented in full in *SI Appendix*, section 2.7.

^§^We provide full results in *SI Appendix*, section 2.2.

^¶^While we believe this norm violation is the most indicative of the particular sort of democratic backsliding associated with election denial, we acknowledge it is likely not perfectly correlated with the belief that the 2020 election was stolen.

^#^In *SI Appendix*, 3, we provide multiple robustness checks to our results, which are all consistent with those presented here.

^‖^We note that we are not sampling or weighting on MAGA-identification, meaning the presence of MAGA-identifiers in our sample is incidental.

^**^Benchmarking this effect size against other political science research requires standardization often not possible without a thorough meta-analysis. One such meta-analysis finds the average positive versus negative standard framing effect on political attitudes in the existing literature to be 0.41, roughly 13 times greater than what our design is powered to detect (62). Other political science research is rarely sufficiently powered to detect such a small effect. Another meta-analysis, for example, finds only 15% of existing studies using regression discontinuity designs are 80% powered to detect standard effect sizes of 0.1 (63).
