# Supplementary material for: Uncommon and nonpartisan: Antidemocratic attitudes in the American public
Source: Proc Natl Acad Sci U S A. 2024 Mar 18;121(13):e2313013121. doi: 10.1073/pnas.2313013121 (PMC10990094; doi:10.1073/pnas.2313013121)
Supplement: Supplementary file 1 — Appendix 01 (PDF) [file pnas.2313013121.sapp.pdf]

Supporting information for Uncommon and Nonpartisan: Anti-Democratic Attitudes In The American Public

Derek E. Holliday[a], Shanto Iyengar[a], Yphtach Lelkes[b], and Sean J. Westwood[c]

[a]Stanford University
[b]University of Pennsylvania
[c]Dartmouth College

February 13, 2024

Contents

1 Summary of Literature **3**

2 Additional Descriptive Results **4**
2.1 Affective Polarization 4
2.2 Scale of Norm Violation Support 6
2.3 Continuous Measure of Anti-democratic Attitudes 7
2.4 Electorally salient demographics: All norms 11
2.5 Survey Toplines 14
2.6 Partisan Differences in Support and Attitudes 16
2.7 Attitudinal Stability 18
2.8 Partisan Strength, Affective Polarization, and Norm Violation Support . . . 22

3 Multilevel Model Robustness **24**

4 Random Forest **28**
4.1 Full Variable Importance 29
4.2 OLS Regressions 32
4.3 Understanding Importance 34

5 Model Selection and Performance **35**
5.1 Why Random Forest? 35
5.2 Model Accuracy 35

6 Social Desirability **38**

7 Survey Characteristics **39**
7.1 Sampling 39
7.2 Weighting 39
7.3 Survey Questions 41
7.3.1 Affect and Trust 41
7.3.2 Democratic Norms 41
7.3.3 Political Violence 42
7.3.4 Political Violence Perceptions 43

List of Figures

S1 Affective polarization by partisanship 4
S2 Affective polarization by strength of partisanship 5
S3 Perception of out-party support for norm violations and political violence by party and strength of partisanship 5
S4 Norm Violations Supported 6
S5 Average support for norm violations (Continuous DV) 7

S6 Average support for norm violations A. by Party and B. Over Time (Continuous DV) . . . 8
S7 Distribution of support for norm violations by party . . . 9
S8 Distribution of latent support for violence by party (left) and estimated difficulty parameters per item (right) . . . 10
S9 Average support for ignoring outparty court decisions by salient electoral demographics . . . 11
S10 Average support for censoring partisan media by salient electoral demographics 12
S11 Average support for reducing ouparty polling stations by salient electoral demographics . . . 13
S12 Norm Support by Affective Polarization (LOESS) . . . 23
S13 Variable importance estimates (demographic predictors), pooled over partisans 30
S14 Variable importance estimates (all predictors), pooled over partisans . . . 31
S15 Model performance: Demographic Predictors . . . 36
S16 Model performance: All Predictors . . . 37
S17 Distribution of Sample Weights . . . 40

List of Tables

S1 Selected Research on American Partisans' Support for Anti-democratic Actions 3
S2 Number of Norm Violations Supported by Party . . . 6
S3 Number of Norm Violations Supported by Party (Excluding Exec. Power) . 6
S4 Survey Toplines . . . 15
S5 Partisan Differences in Support: Norms . . . 16
S6 Partisan Differences in Support: Violence . . . 16
S7 Affective Polarization: Party Differences . . . 17
S8 Support for Democratic Norm Violations by Week (Non-Panel) . . . 18
S9 Support for Political Violence by Week (Non-Panel) . . . 18
S10 Support for Democratic Norm Violations by Week (Panel) . . . 19
S11 Support for Political Violence by Week (Panel) . . . 19
S12 Effect of Lagged Support on Current Support (Democratic Norms) . . . 20
S13 Effect of Lagged Support on Current Support (Violence) . . . 21
S14 Norm Violation Support by Binned Affective Polarization . . . 22
S15 Multilevel Regression Results - Washington Post . . . 24
S16 Multilevel Regression Results - 2023 Only . . . 25
S17 Multilevel Regression Results (Continuous DV) . . . 26
S18 Multilevel Regression Results - Any Denier Candidate for House . . . 27
S19 OLS Predictors of Democratic Norm Violations (Unweighted) . . . 32
S20 OLS Predictors of Political Violence (Unweighted) . . . 33
S21 Social Desirability Regression Results . . . 38

1 Summary of Literature

In Table S1, we provide a tabular summary of existing expectations for and against the presence of partisan asymmetry and provide greater detail of such expectations in the main text.

Table S1: Selected Research on American Partisans' Support for Anti-democratic Actions

**Paper**	**Symmetric**	**Partisan Strength Effect**	**Response to Threat**
Braley et al. (2021)	✓		✓
Graham (2021)	✓	✓	
Graham and Svolik (2020)	✓	✓	
Grossmann and Hopkins (2015)	✓		
Krishnarajan (2023)	✓		
Simonovits, McCoy and Littvay (2022)	✓	✓	✓
Gidengil, Stolle and Bergeron-Boutin (2022)		✓	
Pasek et al. (2022)		✓	✓
Touchton, Klofstad and Uscinski (2023)			

2 Additional Descriptive Results

Here we provide additional descriptive results from our survey not included in the main text of the paper.

2.1 Affective Polarization

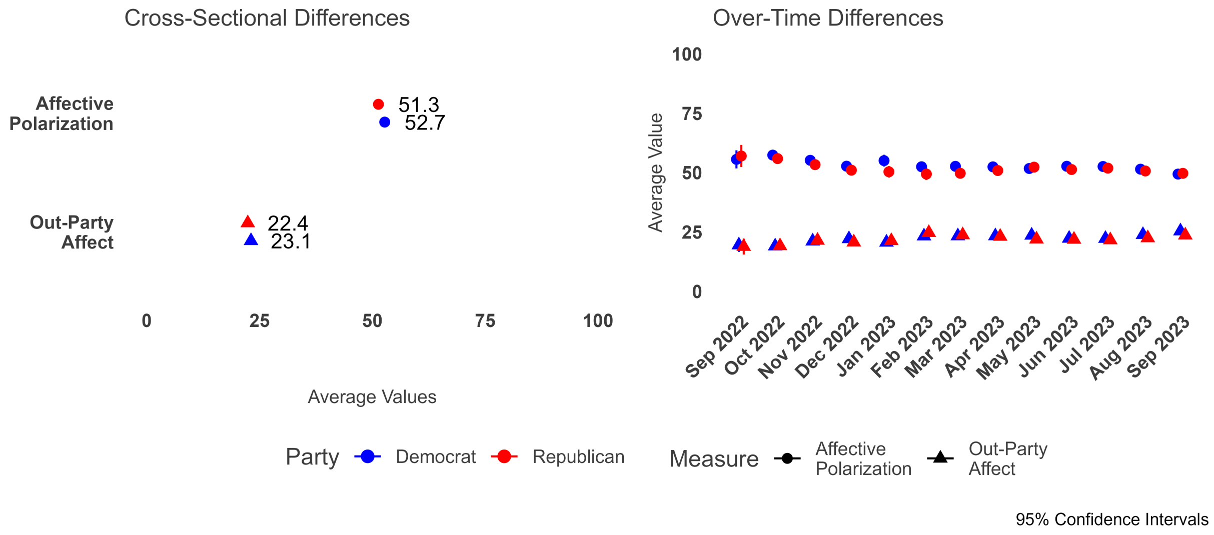

Figure S1: Affective polarization by partisanship

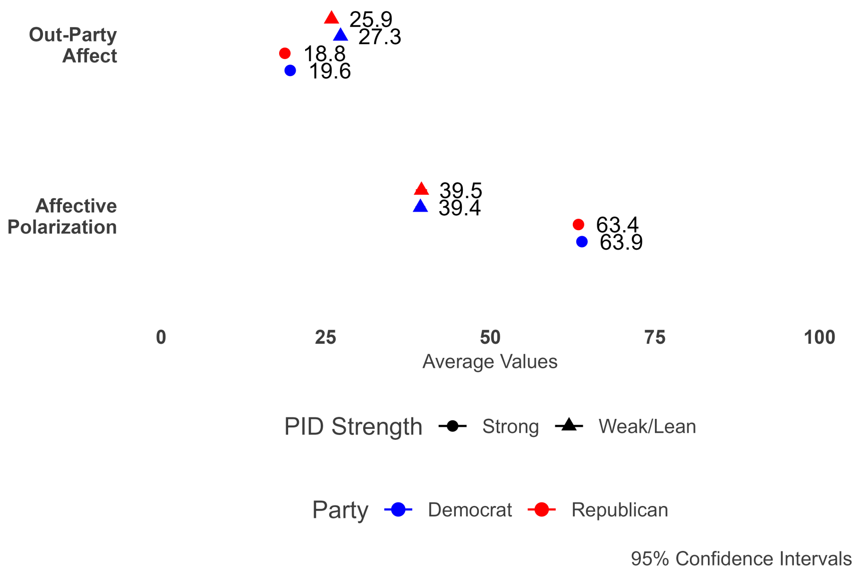

Figure S2: Affective polarization by strength of partisanship

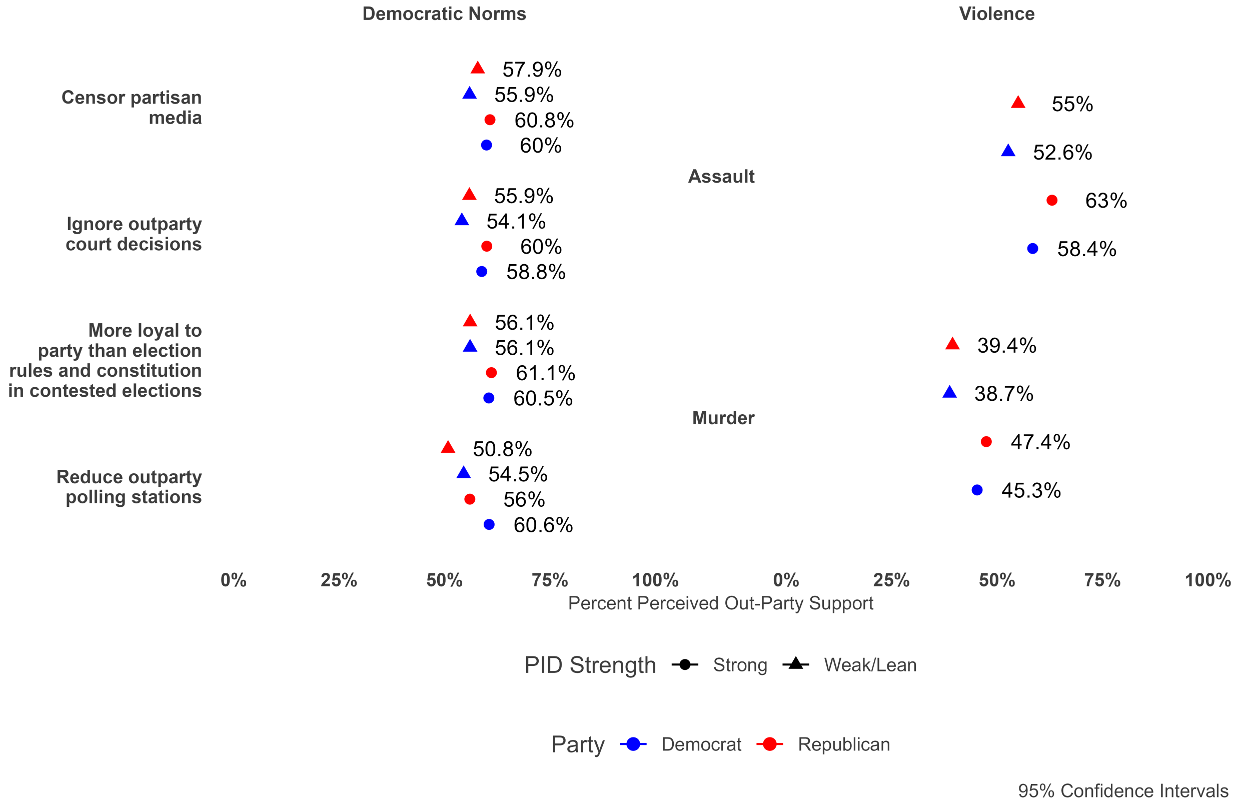

Figure S3: Perception of out-party support for norm violations and political violence by party and strength of partisanship

2.2 Scale of Norm Violation Support

In Table S2, we estimate the percentage of partisans supporting 0 to 5 of the norm violations in our survey. While slightly more Democrats than Republicans support one norm violation, this difference is mostly driven by support for the President circumventing Congress. The parties have almost identical levels of support for two or more violations. We re-estimate results after removing the executive power norm from our results in Table S3. Results are presented graphically in Figure S4.

Table S2: Number of Norm Violations Supported by Party

	Norm Violations Supported (95% confidence intervals)					
Party	0	1	2	3	4	5
Democrat	43.3% [42.5,44.1]	33.8% [33.1,34.6]	11.4% [10.9,11.9]	4.7% [4.4,5.1]	3.4% [3.1,3.7]	3.4% [3.1,3.8]
Republican	55% [54,56]	22.6% [21.8,23.4]	11.1% [10.5,11.7]	5.7% [5.2,6.2]	3% [2.7,3.4]	2.7% [2.3,3]

Table S3: Number of Norm Violations Supported by Party (Excluding Exec. Power)

	Norm Violations Supported (95% confidence intervals)				
Party	0	1	2	3	4
Democrat	69% [68.2,69.7]	17.2% [16.6,17.9]	6% [5.7,6.5]	4% [3.7,4.4]	3.8% [3.4,4.1]
Republican	62.7% [61.7,63.6]	21.6% [20.8,22.4]	9% [8.4,9.6]	3.7% [3.4,4.1]	3% [2.7,3.4]

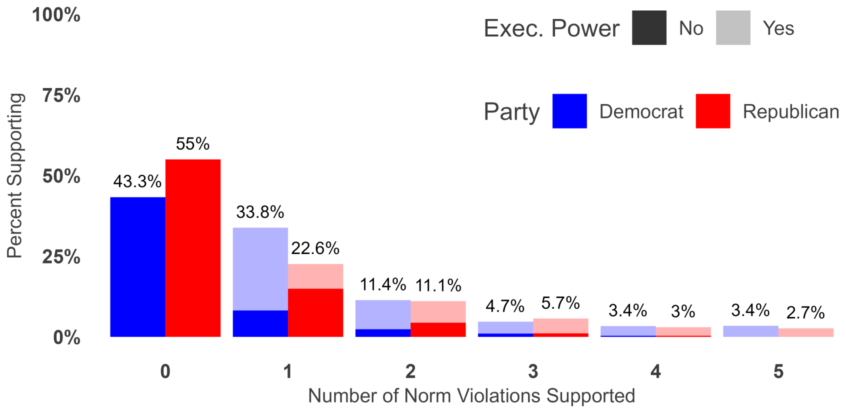

Figure S4: Norm Violations Supported

2.3 Continuous Measure of Anti-democratic Attitudes

While we prefer our binary measures of support in the main manuscript for their clarity, here we present the main results of Figures 1 and 2 in the manuscript, but instead using the continuous operatonalization of our measures for support for norm violations.

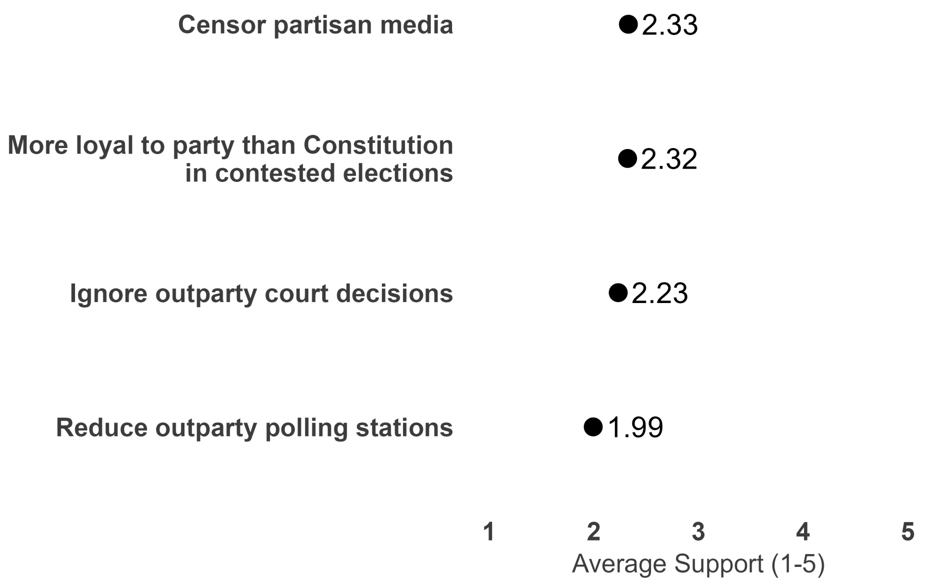

Figure S5: Average support for norm violations (Continuous DV)

We do not present continuous results for political violence, as we cannot compute a direct analogue given how questions of increasing intensity of violence were only shown to respondents conditional on clearing a threshold on the previous item. We can, however, estimate latent support for violence generally by party by fitting a Rasch rating scale model (a class of item response models), replacing missing responses with 0 and allowing the violence item difficulty to vary across items. In theory, this allows for missingness to be controlled for by increasing item difficulty. The results are given in Figure S8. While the absolute numerical values are substantively unimportant, the relative values signal relatively low overall support for political violence and no difference by party.

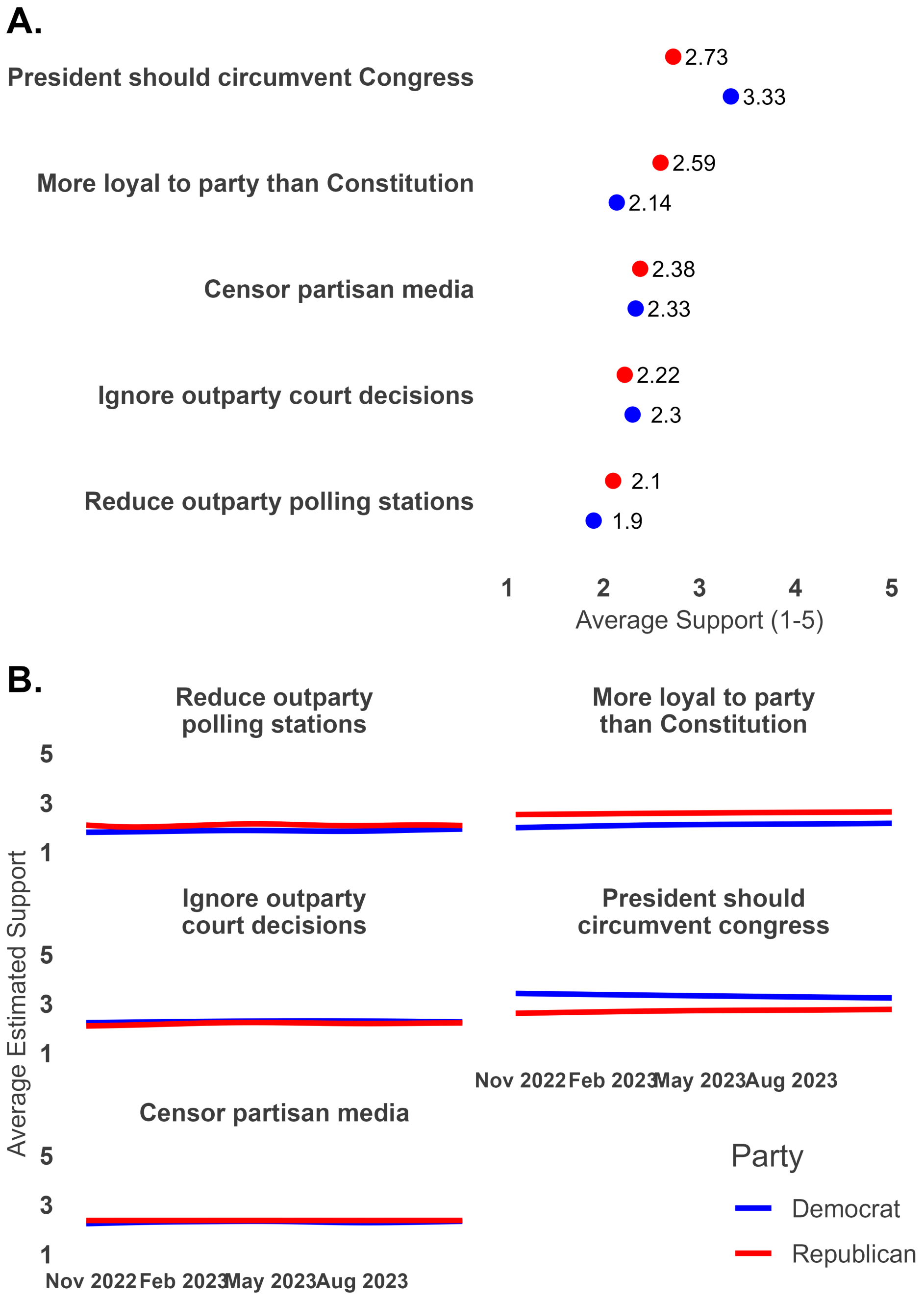

Figure S6: Average support for norm violations A. by Party and B. Over Time (Continuous DV)

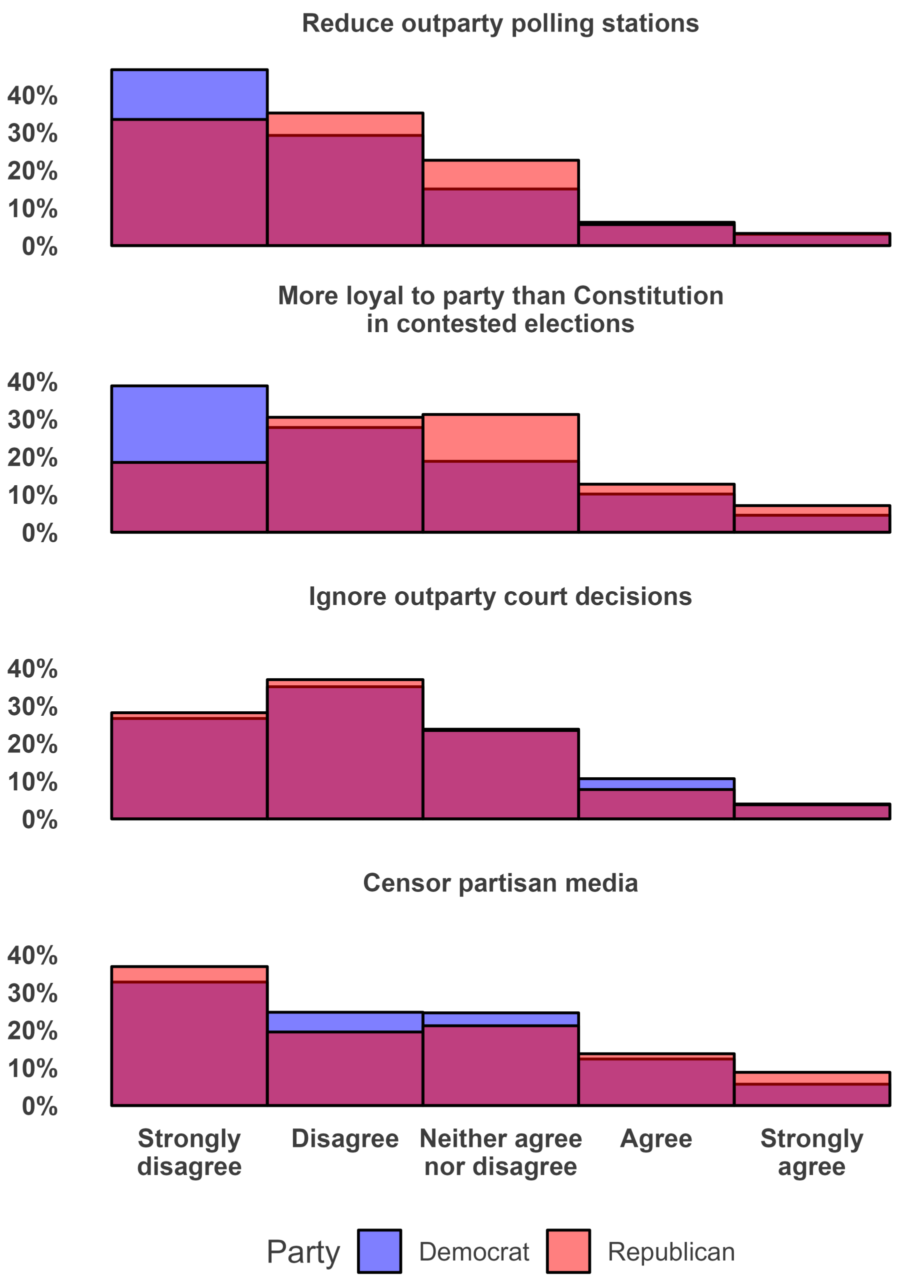

Figure S7: Distribution of support for norm violations by party

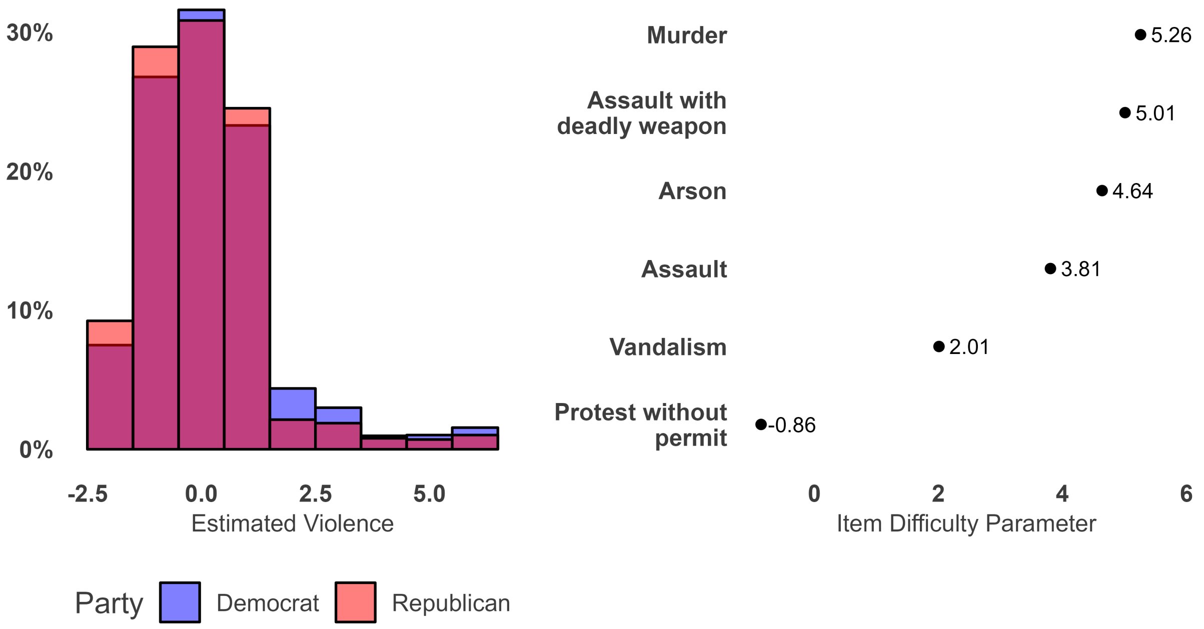

Figure S8: Distribution of latent support for violence by party (left) and estimated difficulty parameters per item (right)

2.4 Electorally salient demographics: All norms

Below we present results mirroring those from Figure 5 in the main manuscript for the remaining norm violations.

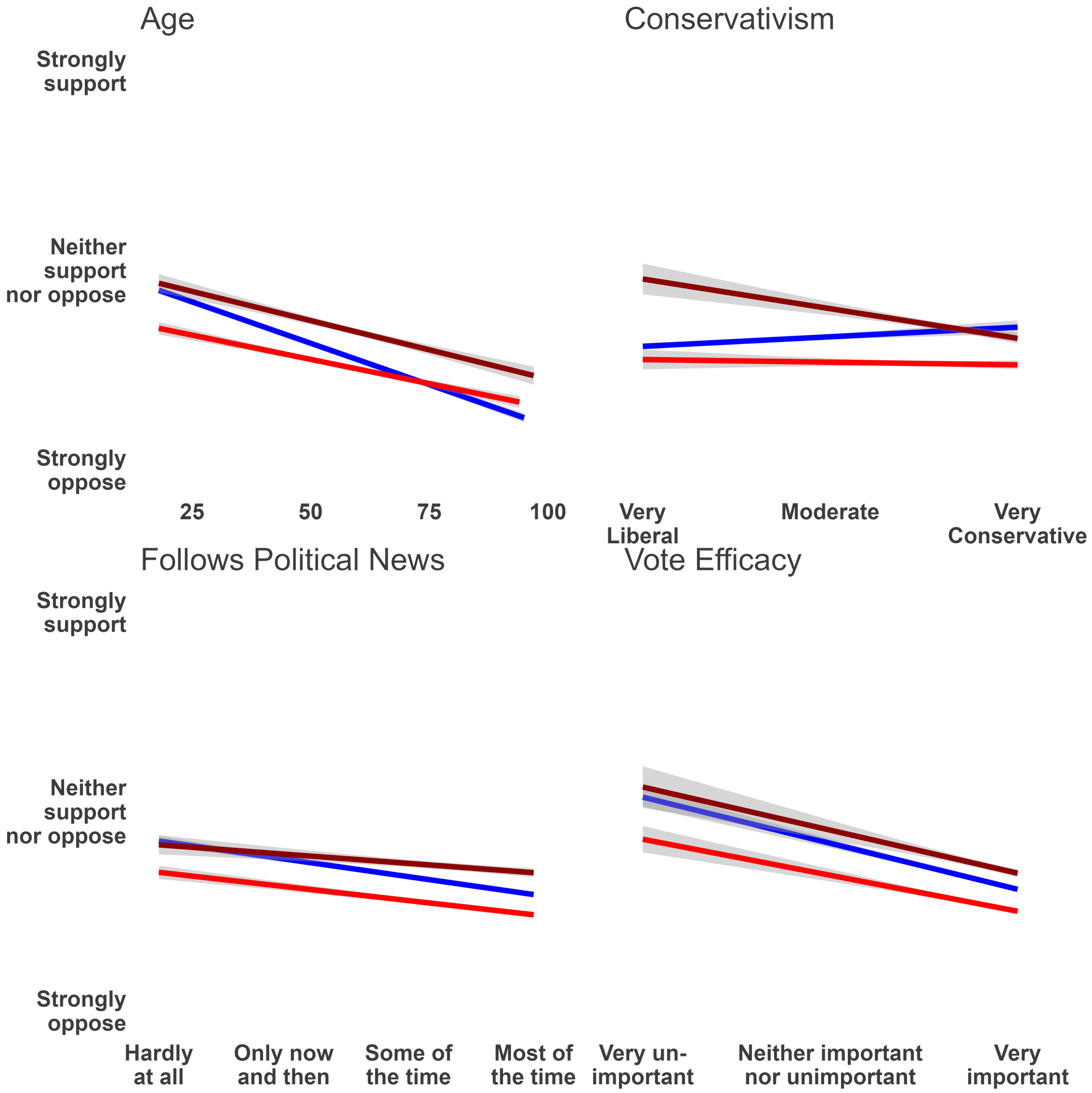

Figure S9: Average support for ignoring outparty court decisions by salient electoral demographics

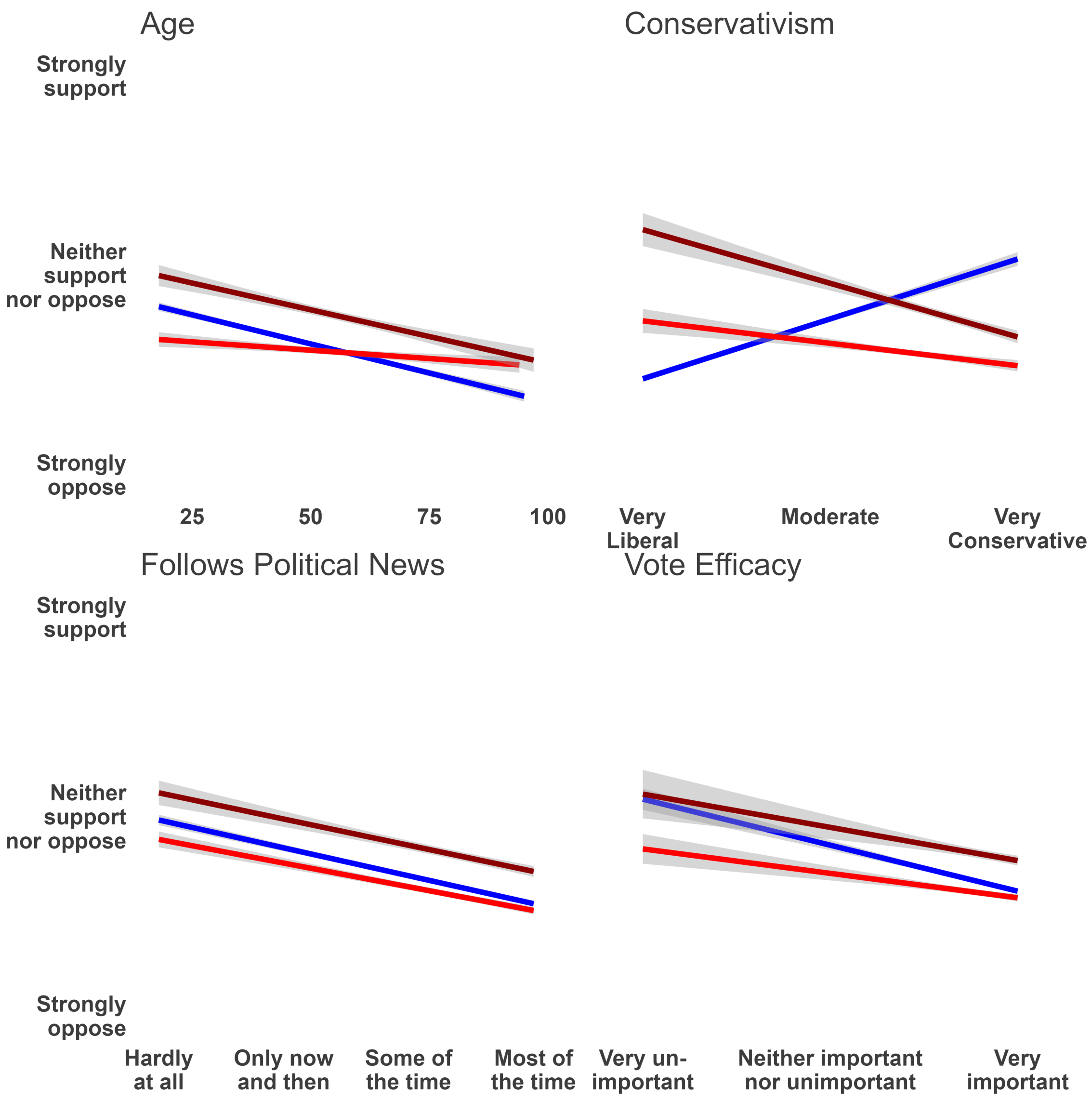

Figure S10: Average support for censoring partisan media by salient electoral demographics

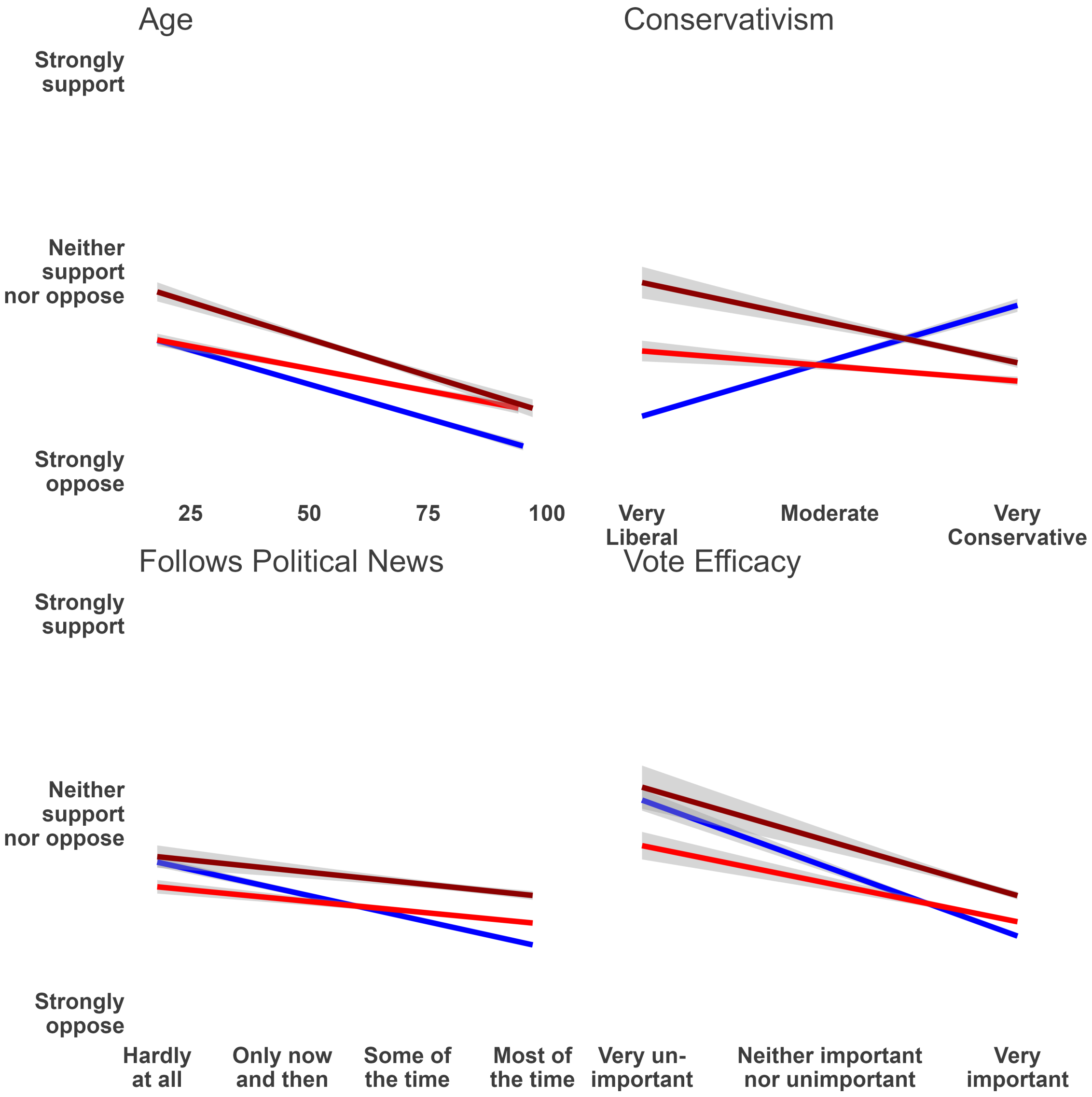

Figure S11: Average support for reducing ouparty polling stations by salient electoral demographics

2.5 Survey Toplines

Table S4: Survey Toplines

	Unweighted N	Survey Weighted Proportion (SE)
**PID**		
Democrat	22168	45.68% (0.26)
Independent	7899	17.01% (0.2)
Republican	15028	37.31% (0.26)
**Race**		
Non-white	15281	35.62% (0.26)
White	29814	64.38% (0.26)
**Education**		
College	15240	31.87% (0.24)
No College	29855	68.13% (0.24)
**Evangelical Status**		
Born again	12784	30.1% (0.25)
Not born again	32311	69.9% (0.25)
**Urban**		
Big city	9078	20.48% (0.22)
Smaller city	7737	16.91% (0.2)
Suburban	15684	34.62% (0.25)
Small town	5604	12.32% (0.17)
Rural	6992	15.66% (0.19)
**Gender**		
Female	23743	50.54% (0.27)
Male	21352	49.46% (0.27)
**Age**		
18-34	11191	27.98% (0.25)
35-64	23322	49.44% (0.26)
65+	10582	22.59% (0.22)
**Income**		
Less than $30K	10448	23.77% (0.23)
$30-$60K	10991	23.94% (0.22)
$60-$100K	9668	21.43% (0.22)
$100-$200K	7511	16.13% (0.19)
¿$200K	1812	3.98% (0.1)
No Response	4665	10.76% (0.17)
**Strength**		
Strong Partisan	19153	42.99% (0.26)
Weak/Lean/Independent	25942	57.01% (0.26)
**Follows Political News**		
Hardly at all	3650	8.42% (0.15)
Only now and then	5746	13.2% (0.18)
Some of the time	12933	29.18% (0.24)
Most of the time	21948	47.24% (0.26)
No Response	818	1.95% (0.08)

2.6 Partisan Differences in Support and Attitudes

Table S5: Partisan Differences in Support: Norms

	Dependent variable:			
	Ignore Courts	Polling Stations	Censorship	Loyalty
	(1)	(2)	(3)	(4)
Republican	−0.031***	−0.004	0.045***	0.052***
	(−0.039, −0.022)	(−0.011, 0.004)	(0.035, 0.056)	(0.042, 0.062)
Constant	0.146***	0.092***	0.180***	0.146***
	(0.140, 0.152)	(0.087, 0.097)	(0.173, 0.186)	(0.140, 0.152)
Observations	36,755	36,749	36,737	36,777
Log Likelihood	−16,218.080	−10,093.840	−22,386.850	−20,033.450
Note:				*p<0.05; **p<0.01; ***p<0.001
				Estimated with survey weights and two-sided tests

Table S6: Partisan Differences in Support: Violence

	Dependent variable:			
	Assault	Arson	Deadly weapon	Murder
	(1)	(2)	(3)	(4)
Republican	−0.010***	−0.010***	−0.009***	−0.009***
	(−0.015, −0.005)	(−0.014, −0.006)	(−0.013, −0.005)	(−0.012, −0.005)
Constant	0.036***	0.029***	0.025***	0.022***
	(0.033, 0.039)	(0.026, 0.032)	(0.022, 0.027)	(0.020, 0.025)
Observations	37,196	37,196	37,196	37,196
Log Likelihood	8,021.718	12,479.430	15,897.140	18,324.820
Note:				*p<0.05; **p<0.01; ***p<0.001
				Estimated with survey weights and two-sided tests

Table S7: Affective Polarization: Party Differences

	Dependent variable:	
	affpol	outparty
	(1)	(2)
Republican	-1.397^{***}	-0.714^{*}
	(-2.226, -0.568)	(-1.323, -0.104)
Constant	52.738^{***}	23.102^{***}
	(52.196, 53.279)	(22.697, 23.508)
Observations	37,075	37,092
Log Likelihood	$-185{,}000.300$	$-173{,}663.900$
Note:		*p<0.05; **p<0.01; ***p<0.001
		Estimated with survey weights and two-sided tests

2.7 Attitudinal Stability

Table S8: Support for Democratic Norm Violations by Week (Non-Panel)

Dependent Variables:	Ignore Courts	Loyalty	Polling Stations	Exec. Power	Censorship
Model:	(1)	(2)	(3)	(4)	(5)
Variables					
Constant	0.12***	0.14***	0.07***	0.32***	0.17***
	(0.003)	(0.003)	(0.003)	(0.004)	(0.004)
week_since	0.0002*	0.0007***	0.0003***	-0.0002	0.0003*
	(9.6×10^{-5})	(0.0001)	(8.1×10^{-5})	(0.0001)	(0.0001)
Fit statistics					
Observations	44,544	44,567	44,530	44,540	44,538
R^2	0.00011	0.00090	0.00041	4.19×10^{-5}	0.00012
Adjusted R^2	8.86×10^{-5}	0.00088	0.00039	1.95×10^{-5}	9.37×10^{-5}

IID standard-errors in parentheses
*Signif. Codes: ***: 0.001, **: 0.01, *: 0.05*

Table S9: Support for Political Violence by Week (Non-Panel)

Dependent Variables:	Assault	Arson	Deadly Weapon	Murder
Model:	(1)	(2)	(3)	(4)
Variables				
Constant	0.02***	0.02***	0.01***	0.01***
	(0.002)	(0.001)	(0.001)	(0.001)
week_since	0.0002***	0.0002***	0.0001***	0.0001***
	(4.8×10^{-5})	(4.2×10^{-5})	(3.9×10^{-5})	(3.6×10^{-5})
Fit statistics				
Observations	45,095	45,095	45,095	45,095
R^2	0.00036	0.00035	0.00032	0.00033
Adjusted R^2	0.00034	0.00033	0.00029	0.00030

IID standard-errors in parentheses
*Signif. Codes: ***: 0.001, **: 0.01, *: 0.05*

Table S10: Support for Democratic Norm Violations by Week (Panel)

Dependent Variables:	Ignore Courts	Loyalty	Polling Stations	Exec. Power	Censorship
Model:	(1)	(2)	(3)	(4)	(5)
Variables					
week_since	-0.0004*	-0.0002	0.0001	-0.0009***	-0.0005*
	(0.0002)	(0.0002)	(0.0001)	(0.0002)	(0.0002)
Fixed-effects					
uid	Yes	Yes	Yes	Yes	Yes
Fit statistics					
Observations	19,259	19,256	19,235	19,253	19,264
R^2	0.69449	0.68796	0.69152	0.69930	0.66598
Within R^2	0.00072	8.48×10^{-5}	0.00013	0.00174	0.00067

Clustered (uid) standard-errors in parentheses
*Signif. Codes: ***: 0.001, **: 0.01, *: 0.05*

Table S11: Support for Political Violence by Week (Panel)

Dependent Variables:	Assault	Arson	Deadly Weapon	Murder
Model:	(1)	(2)	(3)	(4)
Variables				
week_since	3.6×10^{-5}	-4.4×10^{-5}	1.2×10^{-5}	2.5×10^{-5}
	(7.2×10^{-5})	(6.8×10^{-5})	(5.9×10^{-5})	(5.5×10^{-5})
Fixed-effects				
uid	Yes	Yes	Yes	Yes
Fit statistics				
Observations	19,502	19,502	19,502	19,502
R^2	0.65601	0.68246	0.66302	0.66050
Within R^2	1.96×10^{-5}	4.25×10^{-5}	3.84×10^{-6}	1.85×10^{-5}

Clustered (uid) standard-errors in parentheses
*Signif. Codes: ***: 0.001, **: 0.01, *: 0.05*

Table S12: Effect of Lagged Support on Current Support (Democratic Norms)

Dependent Variables:	Ignore Courts	Loyalty	Polling Stations	Exec. Power	Censorship
Model:	(1)	(2)	(3)	(4)	(5)
Variables					
Constant	0.0588***	0.0824***	0.0449***	0.1467***	0.0914***
	(0.0028)	(0.0032)	(0.0024)	(0.0044)	(0.0034)
Ignore Courts (lagged)	0.4895***				
	(0.0079)				
Loyalty (lagged)		0.4740***			
		(0.0081)			
Polling Stations (lagged)			0.4837***		
			(0.0083)		
Exec. Power (lagged)				0.4914***	
				(0.0080)	
Censorship (lagged)					0.4294***
					(0.0081)
Fit statistics					
Observations	11,629	11,639	11,611	11,638	11,641
R^2	0.24823	0.22548	0.22498	0.24602	0.19281
Adjusted R^2	0.24817	0.22541	0.22491	0.24596	0.19274

IID standard-errors in parentheses
*Signif. Codes: ***: 0.001, **: 0.01, *: 0.05*

Table S13: Effect of Lagged Support on Current Support (Violence)

Dependent Variables:	Assault	Arson	Deadly Weapon	Murder
Model:	(1)	(2)	(3)	(4)
Variables				
Constant	0.0163***	0.0110***	0.0095***	0.0085***
	(0.0014)	(0.0011)	(0.0010)	(0.0010)
Assault (lagged)	0.4497***			
	(0.0090)			
Arson (lagged)		0.4763***		
		(0.0088)		
Deadly Weapon (lagged)			0.4460***	
			(0.0088)	
Murder (lagged)				0.4598***
				(0.0089)
Fit statistics				
Observations	11,895	11,895	11,895	11,895
R^2	0.17345	0.19899	0.17641	0.18221
Adjusted R^2	0.17338	0.19892	0.17634	0.18214

IID standard-errors in parentheses
*Signif. Codes: ***: 0.001, **: 0.01, *: 0.05*

2.8 Partisan Strength, Affective Polarization, and Norm Violation Support

Table S14: Norm Violation Support by Binned Affective Polarization

	Dependent variable:			
	Ignore Courts	Polling Stations	Censorship	Loyalty
	(1)	(2)	(3)	(4)
Affpol: 2nd Tercile	-0.028^{***}	-0.051^{***}	-0.029^{***}	-0.042^{***}
	(−0.038, −0.018)	(−0.059, −0.042)	(−0.041, −0.017)	(−0.053, −0.031)
Affpol: 3rd Tercile	0.013^{*}	-0.032^{***}	0.020^{**}	0.009
	(0.002, 0.024)	(−0.042, −0.023)	(0.007, 0.033)	(−0.003, 0.021)
Constant	0.129^{***}	0.108^{***}	0.197^{***}	0.172^{***}
	(0.122, 0.137)	(0.100, 0.115)	(0.188, 0.205)	(0.164, 0.180)
Observations	35,014	35,006	34,996	35,029
Log Likelihood	−14,442.980	−7,821.218	−20,784.100	−18,280.050

Note: *p<0.05; **p<0.01; ***p<0.001
Estimated with survey weights and two-sided tests

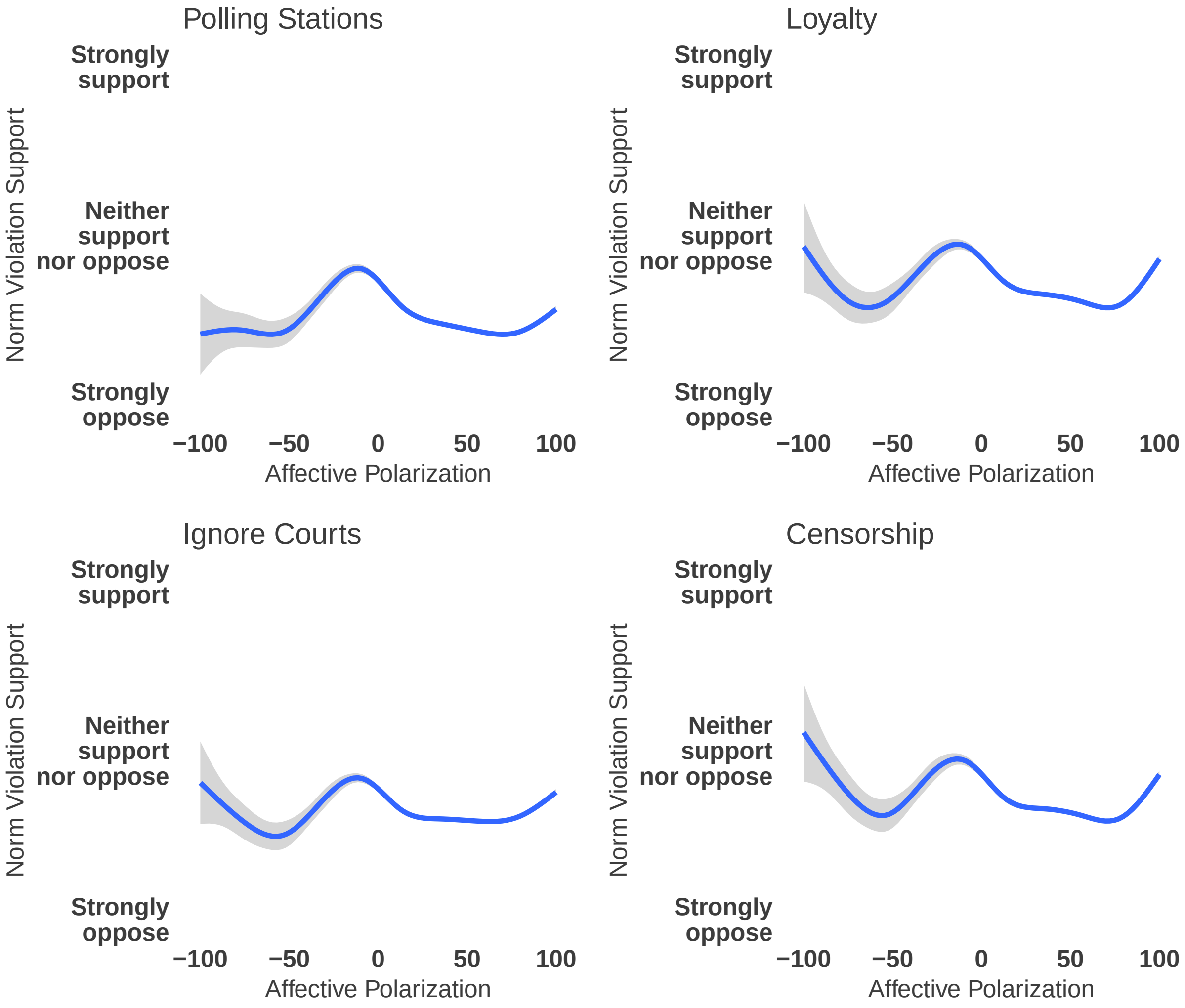

Figure S12: Norm Support by Affective Polarization (LOESS)

3 Multilevel Model Robustness

Table S15: Multilevel Regression Results - Washington Post

	Dependent variable:			
	Loyalty			
	Full Sample		2023 Only	
	(1)	(2)	(3)	(4)
Election Denial (WaPo)	−0.008	−0.022**	−0.010	−0.030**
	(0.006)	(0.008)	(0.007)	(0.009)
Independent		−0.051***		−0.055***
		(0.007)		(0.008)
Republican		0.075***		0.075***
		(0.005)		(0.006)
Election Denial (WaPo):Independent		0.028*		0.039**
		(0.011)		(0.013)
Election Denial (WaPo):Republican		−0.0002		0.008
		(0.009)		(0.010)
Constant	0.144***	0.129***	0.149***	0.136***
	(0.004)	(0.005)	(0.004)	(0.005)
Observations	40,934	40,934	29,790	29,790
Log Likelihood	−14,991.210	−14,749.120	−11,422.900	−11,238.430

Note: *p<0.05; **p<0.01; ***p<0.001
Estimated with survey weights and two-sided tests

Table S16: Multilevel Regression Results - 2023 Only

	Dependent variable:			
	Loyalty			
	(1)	(2)	(3)	(4)
Voted to Overturn	−0.005		−0.021*	
	(0.008)		(0.010)	
Election Denial		−0.003		−0.018
		(0.008)		(0.010)
Independent			−0.049***	−0.050***
			(0.007)	(0.007)
Republican			0.075***	0.076***
			(0.006)	(0.006)
Voted to Overturn:Independent			0.028*	
			(0.014)	
Voted to Overturn:Republican			0.007	
			(0.011)	
Election Denial:Independent				0.031*
				(0.014)
Election Denial:Republican				0.003
				(0.011)
Constant	0.147***	0.147***	0.131***	0.131***
	(0.004)	(0.004)	(0.005)	(0.005)
Observations	29,790	29,790	29,790	29,790
Log Likelihood	−11,423.570	−11,423.720	−11,242.980	−11,242.650
Note:	*p<0.05; **p<0.01; ***p<0.001			
	Estimated with survey weights and two-sided tests			

Table S17: Multilevel Regression Results (Continuous DV)

	Dependent variable:			
	Loyalty (Continuous)			
	(1)	(2)	(3)	(4)
Voted to Overturn	−0.034	0.034		
	(0.024)	(0.029)		
Independent		−0.069***		−0.069***
		(0.020)		(0.020)
Republican		−0.525***		−0.525***
		(0.016)		(0.016)
Voted to Overturn:Independent		−0.050		
		(0.037)		
Voted to Overturn:Republican		0.003		
		(0.030)		
Election Denial:Independent				−0.050
				(0.037)
Election Denial:Republican				0.004
				(0.030)
Election Denial			−0.036	0.029
			(0.023)	(0.028)
Constant	3.760***	3.938***	3.761***	3.939***
	(0.013)	(0.015)	(0.013)	(0.015)
Observations	40,934	40,934	40,934	40,934
Log Likelihood	−61,683.560	−60,954.260	−61,683.410	−60,954.440

Note: *p<0.05; **p<0.01; ***p<0.001
Estimated with survey weights and two-sided tests

Table S18: Multilevel Regression Results - Any Denier Candidate for House

	Dependent variable:	
	Loyalty	
	Full	Full
	(1)	(2)
Election Denier Candidate (WaPo)	−0.008	−0.016*
	(0.006)	(0.008)
Independent		−0.054***
		(0.008)
Republican		0.079***
		(0.006)
Election Denier (WaPo):Independent		0.024*
		(0.011)
Election Denier (WaPo):Republican		−0.007
		(0.009)
Constant	0.145***	0.130***
	(0.005)	(0.005)
Observations	40,934	40,934
Log Likelihood	−14,991.260	−14,750.160
Note:		*p<0.05; **p<0.01; ***p<0.001
		Estimated with survey weights and two-sided tests

4 Random Forest

Partisans endorse democratic norm violations and political violence fairly symmetrically, but are there differences among the various demographic groups that comprise the parties? Because *aggregate* symmetry can mask asymmetry in component groups, we consider the role of demographic-based asymmetries beyond partisanship: age, gender, race/ethnicity, education, religiosity, urban/rural status, and family income. We utilize random forests, a machine-learning approach, to evaluate the importance of different respondent characteristics associated with higher levels of support for democratic norm violations and political violence. Random forests are non-parametric models utilizing an ensemble of classification trees, which allows the detection of non-linear relationships between variables and complex interactions among predictors Breiman (2001).

To preview our results, we show (1) partisan affect to be a consistently strong predictor of support, (2) party identification is a consistently weak predictor of support, and (3) predictors of norm violations behave similarly across partisans.

Here we use random forests, an increasingly popular method in prediction problems in political science Hill and Jones (2014); McAlexander and Mentch (2020); Montgomery and Olivella (2018); Muchlinski et al. (2016); Suzuki (2015). Random forests are non-parametric models utilizing an ensemble of classification trees, which allows for the detection of non-linear relationships between variables and complex interactions among predictors Breiman (2001). Specifically, we fit a series of random forest classification models with binary operationalizations of each of our individual democratic norm and political violence items as dependent variables. We include a large battery of predictors suggested by the literature to be associated with support for democratic norm violations and political violence, including the component measures of affective polarization (out- and in-party affect), party identification and strength of identification, interest in politics, residing in a state with an opposite-party governor, and various demographic variables (age, gender, race/ethnicity, education, religiosity, urban/rural status, and family income).

We prefer such an approach over the typical linear (or logistic) specification for a number of reasons. First, beyond random forest's ability to detect non-linear relationships and complex interactions, they also alleviate multicollinearity concerns endemic to kitchen-sink regressions due to the random sampling of parameters from the feature space. This means that correlations between predictors are not an issue in this approach, so multiple correlated variables can be included in a model.[1]

Machine learning frameworks (beyond random forest) for evaluating variable importance are able to validate predictive claims through the splitting of data into training and testing sets. In typical regression approaches, authors makes claims of predictive importance after fitting a model using the whole dataset. But if the fitted model is never used to predict unseen observations, it is possible (likely, even) the model is overfit to the data. In a

[1]Relatedly, random forests offer a more straightforward approach to determining variable importance in prediction. Beyond well-trod problems with interpretability of control variables (King 1986), regressions with large numbers of under- or un-explained control variables can easily lead to significance of the main variable of interest without proper theoretical justification (Lenz and Sahn 2021). In random forests, the focus on prediction alleviates interpretation concerns; importance is based not on statistical significant but on how often the variable helps make better predictions.

machine-learning framework, models are only as good as the out-of-sample predictions they make. To assess performance of our models, we fit to a randomly split sample of our data and test model performance on the left-out observations.[2]

We keep hyperparameter settings constant across all models for ease of comparability across predictions, which we tune using 10-fold cross-validation with our summary measure of support for democratic norm violations. Specifically, we use $n = 1,000$ trees. We require a minimum of 28 observations in a node for said node to be split further. Additionally, we set the number of predictors randomly sampled at each split to be 4.

Variable importance is calculated using bias-corrected Gini impurity scores, which are robust to inflations in importance in high-cardinality predictors Nembrini, König and Wright (2018).

4.1 Full Variable Importance

[2]We split out data into 80% training data and 20% testing data, stratified by party, and keeping only observations with non-missing data for each of our variables. This yields a training set of 25,802 respondents and a testing set of 6,452 respondents.

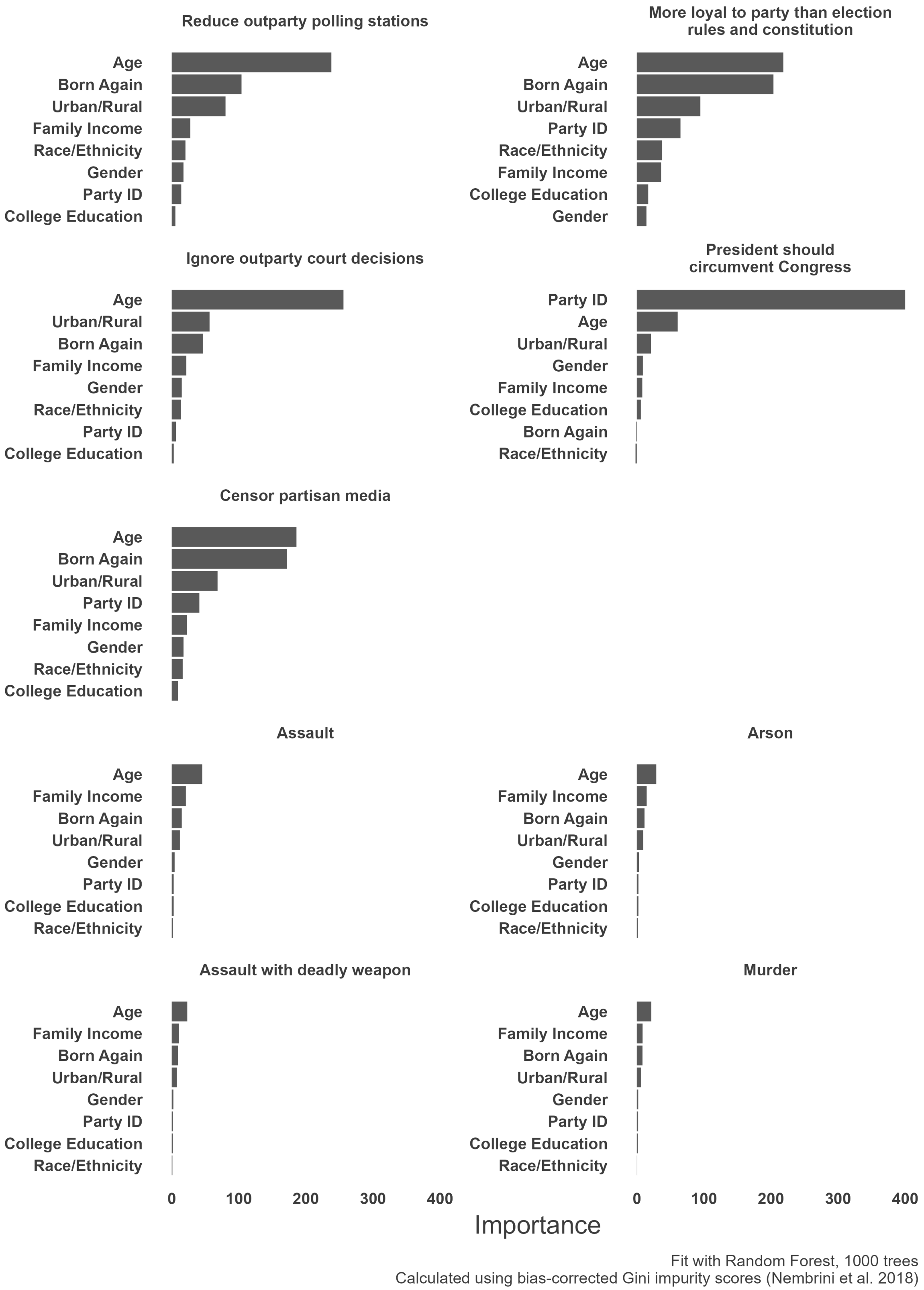

Figure S13: Variable importance estimates (demographic predictors), pooled over partisans

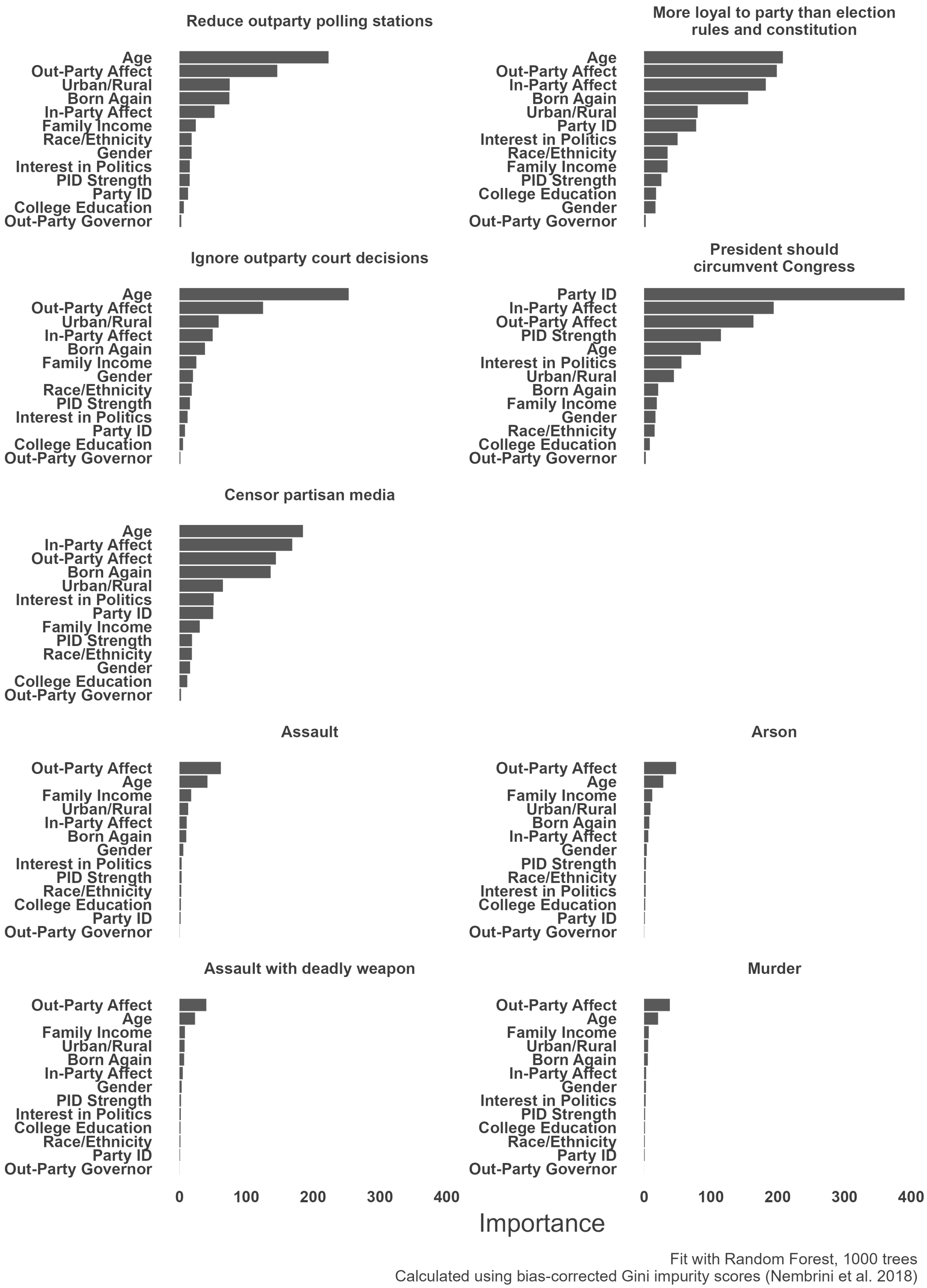

Figure S14: Variable importance estimates (all predictors), pooled over partisans

4.2 OLS Regressions

We present unweighted regression results to more directly compare OLS to our preferred random forest specification, which is also unweighted.

Table S19: OLS Predictors of Democratic Norm Violations (Unweighted)

	Dependent variable:				
	Ignore Courts	Loyalty	Polling Stations	Exec. Power	Censorship
	(1)	(2)	(3)	(4)	(5)
Republican	−0.010*	0.072***	0.008*	−0.182***	0.058***
	(0.004)	(0.004)	(0.003)	(0.006)	(0.005)
White	−0.017***	−0.039***	−0.013***	−0.028***	−0.026***
	(0.004)	(0.004)	(0.003)	(0.006)	(0.005)
College-Educated	0.017***	0.041***	0.019***	0.013*	0.034***
	(0.004)	(0.004)	(0.003)	(0.006)	(0.005)
Born Again	0.051***	0.089***	0.063***	0.008	0.098***
	(0.004)	(0.005)	(0.003)	(0.006)	(0.005)
Urban/Rural - Rural	−0.068***	−0.085***	−0.076***	−0.078***	−0.087***
	(0.006)	(0.007)	(0.005)	(0.009)	(0.008)
Urban/Rural - Small town	−0.064***	−0.083***	−0.073***	−0.067***	−0.086***
	(0.007)	(0.007)	(0.005)	(0.010)	(0.008)
Urban/Rural - Smaller city	−0.054***	−0.088***	−0.074***	−0.046***	−0.081***
	(0.006)	(0.006)	(0.005)	(0.009)	(0.007)
Urban/Rural - Suburban	−0.065***	−0.081***	−0.073***	−0.060***	−0.080***
	(0.005)	(0.006)	(0.004)	(0.007)	(0.006)
Male	0.046***	0.033***	0.039***	0.032***	0.007
	(0.004)	(0.004)	(0.003)	(0.005)	(0.004)
Age	−0.004***	−0.003***	−0.003***	−0.001***	−0.002***
	(0.0001)	(0.0001)	(0.0001)	(0.0002)	(0.0001)
Family Income	−0.002**	−0.003***	−0.001	−0.004***	−0.003***
	(0.001)	(0.001)	(0.0005)	(0.001)	(0.001)
Strong Partisan	0.043***	0.042***	0.037***	0.077***	0.033***
	(0.004)	(0.004)	(0.003)	(0.006)	(0.005)
Interest in Politics	0.015***	−0.015***	0.006**	0.035***	−0.018***
	(0.002)	(0.002)	(0.002)	(0.003)	(0.003)
Opposition Governor	−0.002	0.001	0.001	0.0003	0.002
	(0.002)	(0.002)	(0.001)	(0.003)	(0.002)
In-Party Affect	0.001***	0.002***	0.0005***	0.002***	0.002***
	(0.0001)	(0.0001)	(0.0001)	(0.0001)	(0.0001)
Out-Party Affect	0.001***	0.002***	0.001***	−0.001***	0.001***
	(0.0001)	(0.0001)	(0.0001)	(0.0001)	(0.0001)
Constant	0.252***	0.169***	0.164***	0.312***	0.155***
	(0.011)	(0.012)	(0.009)	(0.016)	(0.013)
Observations	32,254	32,254	32,254	32,254	32,254
R^2	0.075	0.112	0.107	0.085	0.079
Adjusted R^2	0.074	0.112	0.107	0.085	0.079
Residual Std. Error (df = 32237)	0.323	0.348	0.264	0.461	0.378
F Statistic (df = 16; 32237)	163.102***	255.364***	242.613***	188.121***	173.506***
Note:					*p<0.05; **p<0.01; ***p<0.001 Estimated with two-sided tests

Table S20: OLS Predictors of Political Violence (Unweighted)

	Dependent variable:			
	Assault	Arson	Deadly Weapon	Murder
	(1)	(2)	(3)	(4)
Republican	−0.008***	−0.008***	−0.007***	−0.007***
	(0.002)	(0.002)	(0.002)	(0.002)
White	−0.001	−0.003	−0.002	−0.001
	(0.002)	(0.002)	(0.002)	(0.002)
College-Educated	0.003	0.002	0.001	0.002
	(0.002)	(0.002)	(0.002)	(0.002)
Born Again	0.025***	0.022***	0.020***	0.019***
	(0.002)	(0.002)	(0.002)	(0.002)
Urban/Rural - Rural	−0.024***	−0.020***	−0.018***	−0.016***
	(0.003)	(0.003)	(0.003)	(0.003)
Urban/Rural - Small town	−0.028***	−0.024***	−0.020***	−0.019***
	(0.004)	(0.003)	(0.003)	(0.003)
Urban/Rural - Smaller city	−0.027***	−0.023***	−0.020***	−0.018***
	(0.003)	(0.003)	(0.003)	(0.002)
Urban/Rural - Suburban	−0.028***	−0.024***	−0.022***	−0.020***
	(0.003)	(0.002)	(0.002)	(0.002)
Male	0.022***	0.017***	0.016***	0.014***
	(0.002)	(0.002)	(0.002)	(0.001)
Age	−0.001***	−0.001***	−0.001***	−0.001***
	(0.0001)	(0.0001)	(0.00005)	(0.00005)
Family Income	0.001*	0.0005	0.0003	0.0004*
	(0.0003)	(0.0003)	(0.0002)	(0.0002)
Strong Partisan	0.016***	0.012***	0.012***	0.009***
	(0.002)	(0.002)	(0.002)	(0.002)
Interest in Politics	0.006***	0.007***	0.006***	0.005***
	(0.001)	(0.001)	(0.001)	(0.001)
Opposition Governor	−0.001	0.0003	0.0005	0.001
	(0.001)	(0.001)	(0.001)	(0.001)
In-Party Affect	0.00003	0.00003	0.00005	0.0001*
	(0.0001)	(0.00004)	(0.00004)	(0.00004)
Out-Party Affect	0.001***	0.001***	0.001***	0.001***
	(0.00004)	(0.00004)	(0.00003)	(0.00003)
Constant	0.052***	0.037***	0.029***	0.021***
	(0.006)	(0.005)	(0.005)	(0.004)
Observations	32,254	32,254	32,254	32,254
R^2	0.057	0.053	0.051	0.050
Adjusted R^2	0.056	0.052	0.051	0.049
Residual Std. Error (df = 32237)	0.169	0.149	0.136	0.128
F Statistic (df = 16; 32237)	121.375***	112.359***	108.551***	105.623***

Note: *p<0.05; **p<0.01; ***p<0.001
Estimated with two-sided tests

4.3 Understanding Importance

Impurity importance is calculated as the decrease in impurity between a node and its two child nodes for any given variable summed across all trees. As an example, assume a set of 10 respondents split evenly between those who do and don't support at least one democratic norm violation. This node has a Gini impurity of $\frac{5}{10} \times (1 - \frac{5}{10}) + \frac{5}{10} \times (1 - \frac{5}{10}) = 0.5$. If a splitting variable were to then divide that parent node into two child nodes, each with 4 correctly-classified respondents and 1 incorrectly-classified, the new Gini impurity is $\frac{4}{5} \times (1 - \frac{4}{5}) + \frac{1}{5} \times (1 - \frac{1}{5}) = 0.32$, making the reduction in Gini impurity $0.5 - 0.32 = 0.18$. Assuming this is the average across all $1,000$ trees, the importance score would be 180.[3] It is important to note, however, that the different dependent variables specifications we utilize begin with differing baseline levels of impurity, so variable importance should be interpreted *relative* to other variables in the model. Cross-model comparisons can still be made for the overall magnitude of impurity reductions, but identical magnitudes can have different relative model-specific importance.

Of course, variable importance is not a silver bullet for unobserved confounders, and should in no way be interpreted as causal. Just as coefficient significance for any given variable in linear regression could indicate a relationship between the dependent variable and some unobserved factor correlated with the observed predictor, variable importance is just one of many tools to help us triangulate sources of variation in our variables of interest.

[3]Note this is a simplified example. Child nodes are not necessarily of equal size or equal impurity, so Gini impurity is generally calculated as a weighted sum across nodes. Furthermore, the cardinality bias correction from Nembrini, König and Wright (2018) is not present in this toy example.

5 Model Selection and Performance

5.1 Why Random Forest?

Beyond the main reason presented above, we have numerous other reasons to prefer random forest over OLS. Namely, even if a regression coefficient is both large and has low uncertainty, such an estimate is conditional on the full model specification and is vulnerable to collinearity with other predictors, especially with high numbers of predictors Lenz and Sahn (2021). Random forest avoids this vulnerability by generating predictions from an ensemble of different tree-based models, where different sets of predictors are used at different tree depths. In that sense, the role of any single predictor is never "fixed" but allowed to vary based on interactions with other predictors.

If one were to analyze the results of and OLS approach (presented in SI section 4), it would be tempting to come to the conclusion that party identification is an important predictor of support for anti-democratic behavior, as we erroneously tend to interpret our main independent variables independent of scale or other predictors in the regression King (1986). Our approach yields measure of predictive importance robust to multicollinearity, which is most evident in our ability to distinguish between the predictive power of in-party and out-party affect.

5.2 Model Accuracy

In the main text, we showed partisanship poorly differentiates support for democratic norm violations and political violence and that, despite some differences in demographic predictors, the main predictors of both partisan groups' support for such norm violations are the component parts of affective polarization. But while those predictors may be internally important to each model and similar across models, how do those models perform when applied to unseen data, and do they perform similarly? In Figures S15 and S16, we evaluate the performance of the pooled, Democratic, and Republican models with regard to both predictive accuracy and area under the receiver operating characteristic curve (ROC AUC) after applying our fitted models to the held-out testing data of 6,452 respondents, using both demographic and demographic and attitudinal predictors (respectively).

The left panel of Figures S15 and S16 give the accuracy of the models, or the total correct classifications divided by the total number of testing cases. Across all models and all partisan groupings, our predictions perform significantly better than the null model of random guessing (accuracy = 50%). This suggests our models, in which the components of affective polarization were strong predictors, are overall good predictors of support for norm violations. The similarity in accuracy between parties is equally important; the same variables are used in each model, and those same variables yield very similar predictive accuracy, even if they function in slightly different ways. Finally, we note the lack of predictive accuracy gained in the pooled model when adding party identification as a predictor variable. Despite this additional predictor, there is no appreciable difference in the accuracy of the pooled models against the partisan models.

Of course, accuracy can be achieved in a variety of ways. While some of our accuracy scores suggest a well-fitted model able to split hairs between borderline cases, others gain

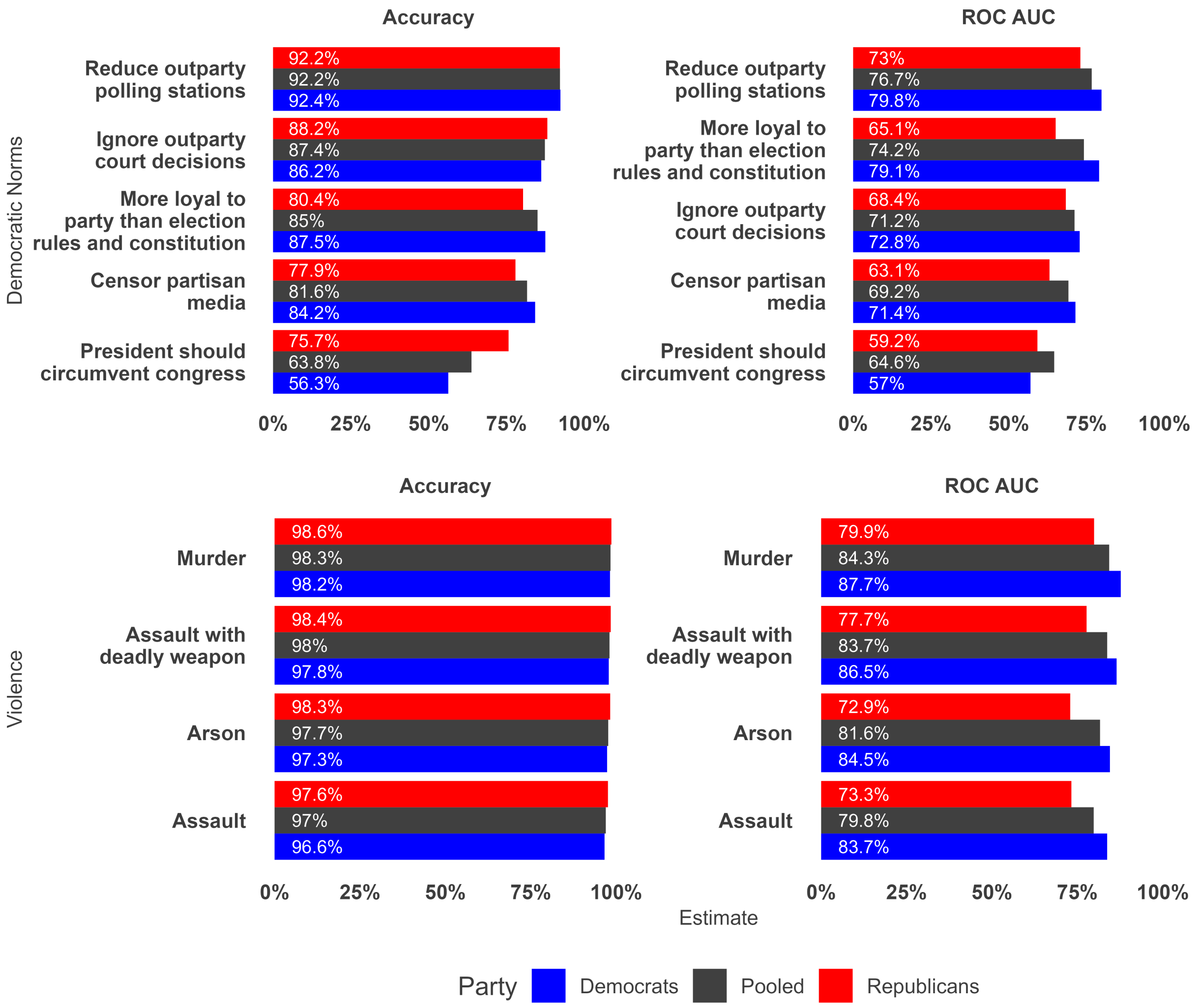

Figure S15: Model performance: Demographic Predictors

accuracy by simply always predicting the most common response. This is exactly the case in many of our political violence models. Since support for political violence is exceptionally rare, it is unsurprising that in all political violence models the random forest models predicted 0 respondents supported violence.

This problem with accuracy in the case of rare cases motivates the inclusion of ROC AUC. The better the model performance, the greater the area under the resulting curve, which is operationalized as the AUC.[4] When looking at ROC AUC (right panel of Figures S15 and S16), performance is generally consistent between partisan groups, although with greater discrepancies than the accuracy measures.

The models for Democratic respondents on average outperform the models for Republican respondents. Still, one would classify both of these models are very capable in their ability

[4]Because the AUC is a function of a trade-off between specificity and sensitivity, the resulting estimate is more robust to rare class problems and a better indicator of overall model performance.

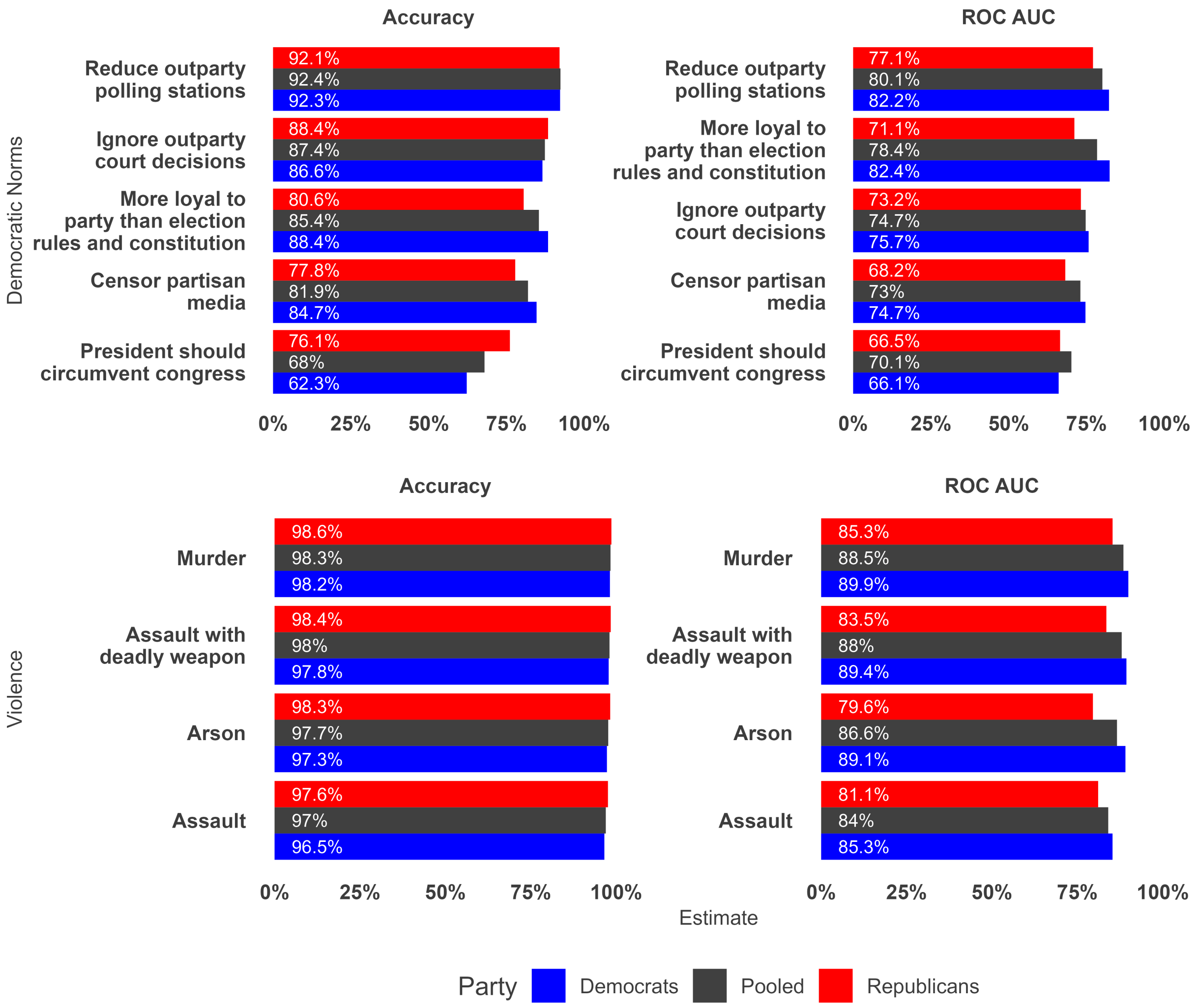

Figure S16: Model performance: All Predictors

to distinguish between those who do and don't support such a democratic norm. Finally, the addition of the party identification variable in the pooled model has negligible impact in overall predictive power.

6 Social Desirability

We consider the possibility that fleeting support for norm violations and political violence could in part be a product of social desirability bias. In a follow-up survey fielded between October 13 and October 19, 2023 ($n = 1{,}000$), we ask the same battery of norm violation and political violence questions, and also embed the 13-item Marlowe-Crowne Social Desirability Scale. We regress total support for norm violations and political violence (measured as the sum of "Agree/Support" responses) and a binary measure of *any* support for norm violations or political violence on the resulting social desirability score (0-13) in the table below. We find no connection between social desirability and reported support for norm violations or political violence.

Table S21: Social Desirability Regression Results

	Dependent variable:			
	Norms (Sum)	Norms (Any)	Violence (Sum)	Violence (Any)
Social Desirability	−0.014	0.002	−0.013	−0.004
	(0.012)	(0.006)	(0.008)	(0.002)
Constant	0.765***	0.336***	0.258***	0.078***
	(0.104)	(0.043)	(0.073)	(0.020)
Observations	1,000	1,000	1,000	1,000
Log Likelihood	−1,574.517	−724.119	−1,192.816	74.375
Note:				*p<0.05; **p<0.01; ***p<0.001
				Estimated with survey weights and two-sided tests

7 Survey Characteristics

7.1 Sampling

The data from this study comes YouGov. The population under study was the adult U.S. population. YouGov employs a two-step process to conduct surveys. Initially, they select a random set of anonymous cases from the American Community Survey (ACS) Public Use Microdata Sample, a reliable data source which accurately represents various variables in the target population. This subset is known as the synthetic sampling frame (SSF) and serves as a model for the final survey sample. For each participant who takes the YouGov survey, they find a matching case within the SSF that shares similar characteristics.

Specifically, YouGov constructs its sampling frame based upon ACS targest for age, gender, race, and education, with weights constructed using propensity scores.[5] YouGov uses nonprobability quota sampling to achieve representativeness, and due to the opt-in design and wide availability of YouGov surveys across many online channels, it is impossible to provide survey response rates.

7.2 Weighting

YouGov respondents are weighted to nationally representative targets for age, gender, race, education, region, political party affiliation, and income level using propensity scores. Large weights are trimmed. Final weights are normalized to sample size. The final distribution of weights is given in Figure S17.

Because a healthy proportion of our sample is panelists, we incorporate a weighting adjustment when calculating cross-sectional results. We divide the final weight (calculated on a week-to-week basis) by the number of times the respondent appears in our sample. We then cluster our standard errors at the respondent level.

[5] See `https://today.yougov.com/about/panel-methodology`

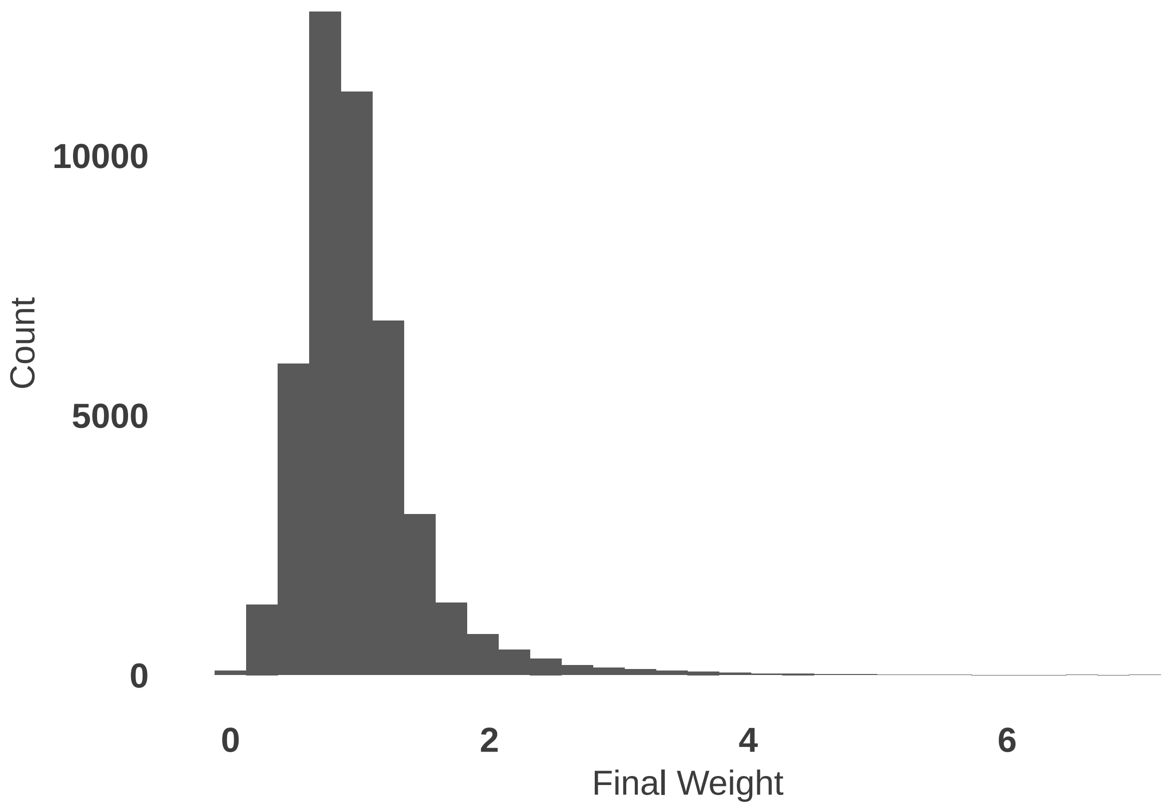

Figure S17: Distribution of Sample Weights

7.3 Survey Questions

Below we include the full question text of the items underlying out estimates, by section. All sections are presented in random order, with the exception of political violence perceptions, which always follow the political violence section. All demographic items were gathered separately by YouGov.

7.3.1 Affect and Trust

Order within this section is completely randomized.

- **democrat_therm:** We'd like you to rate how you feel towards some groups on a scale of 0 to 100. Zero means very unfavorable and 100 means very favorable. Fifty means you do not feel favorable or unfavorable. How would you rate your feeling toward Democrats? [Response: 0-100 thermometer]
- **republican_therm:** We'd like you to rate how you feel towards some groups on a scale of 0 to 100. Zero means very unfavorable and 100 means very favorable. Fifty means you do not feel favorable or unfavorable. How would you rate your feeling toward Republicans? [Response: 0-100 thermometer]
- **vote_importance:** How important or unimportant is it to vote in every election? [Response: Very important / Important / Neither important nor unimportant / Unimportant / Very unimportant]
- **maga:** Would you describe yourself as a... [Response: MAGA Republican / Never Trumper / Neither]

7.3.2 Democratic Norms

Order within this section is randomized, except that perceptions always follow the norms questions. Response options for the norms questions are always Strongly agree / Agree / Neither agree nor disagree / Disagree / Strongly disagree. For perceptions, the response option is a 0-100 slider.

- **norm_polling:** Do you agree or disagree: (inparty) should reduce the number of polling stations in areas that typically support (outparty).
- **norm_polling_perception:** What percent of (outparty) voters do you think agree with the following: (outparty) should reduce the number of polling stations in areas that typically support (inparty).
- **norm_loyalty:** Do you agree or disagree with the following: When a (inparty) candidate questions the outcome of an election other (inparty) should be more loyal to the (inparty) party than to election rules and the constitution.

- **norm_loyalty_perception:** What percent of (outparty) voters do you think agree with the following: When a (inparty) candidate questions the outcome of an election other (inparty) should be more loyal to the (inparty) party than to election rules and the constitution.

- **norm_judges:** Do you agree or disagree: (inparty) elected officials should sometimes consider ignoring court decisions when the judges who issued those decisions were appointed by (outparty) presidents.

- **norm_judges_perception:** What percent of (outparty) voters do you think agree with the following: (inparty) elected officials should sometimes consider ignoring court decisions when the judges who issued those decisions were appointed by (outparty) presidents.

- **norm_censorship:** Do you agree or disagree with the following: The government should be able to censor media sources that spend more time attacking (inparty) than (outparty).

- **norm_censorship_perception:** What percent of (outparty) voters do you think agree with the following: The government should be able to censor media sources that spend more time attacking (inparty) than (outparty).

- **norm_executive:** Do you agree or disagree: If a (inparty) president can't get cooperation from (outparty) members of congress to pass new laws, the (inparty) president should circumvent Congress and issue executive orders on their own to accomplish their priorities.

- **norm_executive_perception:** What percent of (outparty) voters do you think agree with the following: If a (inparty) president can't get cooperation from (outparty) members of congress to pass new laws, the (inparty) president should circumvent Congress and issue executive orders on their own to accomplish their priorities.

7.3.3 Political Violence

The questions in this section appear in the order presented below. For all items, the response options are Strongly support / Support / Neither support nor oppose / Oppose / Strongly oppose.

- **violence1:** (name) was convicted of protesting without a permit. He was arrested by police after leading a protest against (outparty) on the grounds of the county courthouse. He made no effort to acquire the necessary permit for the protest and refused to leave when asked by police. Do you support or oppose (name)'s actions?

- **violence2:** (name) was convicted of vandalism. He was arrested by police after he vandalized several large signs expressing support for candidates of the (outparty) party. Do you support or oppose (name)'s actions?

- **violence3:** (name) was convicted of assault. He was arrested by police for throwing rocks at peaceful (outparty) protesters. Although no one was seriously injured, paramedics bandaged a man with a head wound. Do you support or oppose (name)'s actions?
- **violence4:** (name) was convicted of arson. He was arrested by police as he attempted to run from a fire he started at the local (outparty) headquarters. Although he waited for the building to close for the night, several adjacent buildings were still occupied. Do you support or oppose (name)'s actions?
- **violence5:** (name) was convicted of assault with a deadly weapon. He was arrested by police after driving his car into a crowd of (outparty) protesters. Although no one was killed, several individuals were seriously injured and one spent a month in the hospital. Do you support or oppose (name)'s actions?
- **violence6** (name) was convicted of murder. He was arrested by police after surveillance footage was found showing him stabbing a prominent (outparty) to death. (name) targeted the victim because he believed the victim had prevented him from voting in the last election as part of a conspiracy to stop (inparty) voters. Do you support or oppose (name)'s actions?

7.3.4 Political Violence Perceptions

The following items were shown in a fixed order after the political violence items. The response option for all was a 0-100 slider.

- **violence3_perception:** (name) was convicted of assault. He was arrested by police for throwing rocks at peaceful (inparty) protesters. Although no one was seriously injured, paramedics bandaged a man with a head wound. What percent of (outparty) voters do you think support (names)'s actions?
- **violence6_perception:** (name) was convicted of murder. He was arrested by police after surveillance footage was found showing him stabbing a prominent (inparty) to death. (name) targeted the victim because he believed the victim had prevented him from voting in the last election as part of a conspiracy to stop (outparty) voters. What percent of (outparty) voters do you think support (name)'s actions?

References

Braley, Alia, Gabriel Lenz, Dhaval Adjodah, Hossein Rahnama and Alex Pentland. 2021. "The Subversion Dilemma: Why Voters Who Cherish Democracy Participate in Democratic Backsliding.".
URL: *https://osf.io/my987/*

Breiman, Leo. 2001. "Random Forests." *Machine Learning* 45(1):5–32.
URL: *https://doi.org/10.1023/A:1010933404324*

Gidengil, Elisabeth, Dietlind Stolle and Olivier Bergeron-Boutin. 2022. “The partisan nature of support for democratic backsliding: A comparative perspective.” *European Journal of Political Research* 61(4):901–929. _eprint: https://onlinelibrary.wiley.com/doi/pdf/10.1111/1475-6765.12502.
URL: *https://onlinelibrary.wiley.com/doi/abs/10.1111/1475-6765.12502*

Graham, Matthew H. 2021. “Does Partisan Identity Reduce Support for Electoral Fairness?”.

Graham, Matthew H. and Milan W. Svolik. 2020. “Democracy in America? Partisanship, Polarization, and the Robustness of Support for Democracy in the United States.” *American Political Science Review* 114(2):392–409. Publisher: Cambridge University Press.
URL: *https://www.cambridge.org/core/journals/american-political-science-review/article/democracy-in-america-partisanship-polarization-and-the-robustness-of-support-for-democracy-in-the-united-states/C7C72745B1AD1FF9E363BBFBA9E18867*

Grossmann, Matt and David A. Hopkins. 2015. “Ideological Republicans and Group Interest Democrats: The Asymmetry of American Party Politics.” *Perspectives on Politics* 13(1):119–139. Publisher: [American Political Science Association, Cambridge University Press].
URL: *https://www.jstor.org/stable/43866603*

Hill, Daniel W. and Zachary M. Jones. 2014. “An Empirical Evaluation of Explanations for State Repression.” *American Political Science Review* 108(3):661–687. Publisher: Cambridge University Press.
URL: *https://www.cambridge.org/core/journals/american-political-science-review/article/an-empirical-evaluation-of-explanations-for-state-repression/88E974BACEE4FCF803047599A3DF3A14*

King, Gary. 1986. “How Not to Lie with Statistics: Avoiding Common Mistakes in Quantitative Political Science.” *American Journal of Political Science* 30(3):666–687. Publisher: [Midwest Political Science Association, Wiley].
URL: *https://www.jstor.org/stable/2111095*

Krishnarajan, Suthan. 2023. “Rationalizing Democracy: The Perceptual Bias and (Un)Democratic Behavior.” *American Political Science Review* 117(2):474–496. Publisher: Cambridge University Press.
URL: *https://www.cambridge.org/core/journals/american-political-science-review/article/rationalizing-democracy-the-perceptual-bias-and-undemocratic-behavior/C78EB8AE1CC777B4392EE73727F4F25C*

Lenz, Gabriel S. and Alexander Sahn. 2021. “Achieving Statistical Significance with Control Variables and Without Transparency.” *Political Analysis* 29(3):356–369. Publisher: Cambridge University Press.
URL: *https://www.cambridge.org/core/journals/political-analysis/article/achieving-statistical-significance-with-control-variables-and-without-transparency/1E867C357835019E0C9322B918414045*

McAlexander, Richard J. and Lucas Mentch. 2020. "Predictive inference with random forests: A new perspective on classical analyses." *Research & Politics* 7(1):2053168020905487. Publisher: SAGE Publications Ltd.
URL: *https://doi.org/10.1177/2053168020905487*

Montgomery, Jacob M. and Santiago Olivella. 2018. "Tree-Based Models for Political Science Data." *American Journal of Political Science* 62(3):729–744. Publisher: [Midwest Political Science Association, Wiley].
URL: *https://www.jstor.org/stable/26598778*

Muchlinski, David, David Siroky, Jingrui He and Matthew Kocher. 2016. "Comparing Random Forest with Logistic Regression for Predicting Class-Imbalanced Civil War Onset Data." *Political Analysis* 24(1):87–103. Publisher: [Oxford University Press, Society for Political Methodology].
URL: *https://www.jstor.org/stable/24573207*

Nembrini, Stefano, Inke R König and Marvin N Wright. 2018. "The revival of the Gini importance?" *Bioinformatics* 34(21):3711–3718.
URL: *https://doi.org/10.1093/bioinformatics/bty373*

Pasek, Michael H., Lee-Or Ankori-Karlinsky, Alex Levy-Vene and Samantha L. Moore-Berg. 2022. "Misperceptions about out-partisans' democratic values may erode democracy." *Scientific Reports* 12(1):16284. Number: 1 Publisher: Nature Publishing Group.
URL: *https://www.nature.com/articles/s41598-022-19616-4*

Simonovits, Gabor, Jennifer McCoy and Levente Littvay. 2022. "Democratic Hypocrisy and Out-Group Threat: Explaining Citizen Support for Democratic Erosion." *The Journal of Politics* 84(3):1806–1811. Publisher: The University of Chicago Press.
URL: *https://www.journals.uchicago.edu/doi/full/10.1086/719009*

Suzuki, Akisato. 2015. "Is more better or worse? New empirics on nuclear proliferation and interstate conflict by Random Forests." *Research & Politics* 2(2):2053168015589625. Publisher: SAGE Publications Ltd.
URL: *https://doi.org/10.1177/2053168015589625*

Touchton, Michael, Casey Klofstad and Joseph Uscinski. 2023. "Does partisanship promote anti-democratic impulses? Evidence from a survey experiment." *Journal of Elections, Public Opinion and Parties* 33(2):197–209. Publisher: Routledge _eprint: https://doi.org/10.1080/17457289.2020.1844218.
URL: *https://doi.org/10.1080/17457289.2020.1844218*
